# Rapid depletion and super-resolution microscopy reveal dual roles of SRSF5 in coordinating nuclear speckle–paraspeckle crosstalk during cellular stress

**DOI:** 10.1093/nar/gkaf713

**Published:** 2025-07-26

**Authors:** Ellen Kazumi Okuda, Laurell Fridolin Kessler, Benjamin Arnold, Ricarda J Riegger, Maria Clara Hernández Cañás, Ewelina Zebrowska, Cem Bakisoglu, Mara Rudigier, Christine Krost, Helder Y Nagasse, Jan Keiten-Schmitz, Stefan Müller, David Stanek, Dorothee Dormann, Kathi Zarnack, Mike Heilemann, Michaela Müller-McNicoll

**Affiliations:** Institute of Molecular Biosciences, Goethe University, 60438 Frankfurt am Main, Germany; IMPRS on Cellular Biophysics, 60438 Frankfurt am Main, Germany; Institute of Physical and Theoretical Chemistry, Goethe University, 60438 Frankfurt am Main, Germany; Institute of Molecular Biosciences, Goethe University, 60438 Frankfurt am Main, Germany; Institute of Molecular Biosciences, Goethe University, 60438 Frankfurt am Main, Germany; Institute of Molecular Biosciences, Goethe University, 60438 Frankfurt am Main, Germany; Buchmann Institute for Molecular Life Sciences (BMLS), 60438 Frankfurt am Main, Germany; Institute of Molecular Biosciences, Goethe University, 60438 Frankfurt am Main, Germany; Institute of Molecular Biosciences, Goethe University, 60438 Frankfurt am Main, Germany; Buchmann Institute for Molecular Life Sciences (BMLS), 60438 Frankfurt am Main, Germany; Institute of Molecular Biosciences, Goethe University, 60438 Frankfurt am Main, Germany; Institute of Molecular Biosciences, Goethe University, 60438 Frankfurt am Main, Germany; Institute of Molecular Biosciences, Goethe University, 60438 Frankfurt am Main, Germany; Institute of Biochemistry II, Goethe University Frankfurt, 60528 Frankfurt am Main, Germany; Institute of Biochemistry II, Goethe University Frankfurt, 60528 Frankfurt am Main, Germany; Institute of Molecular Genetics (IMG), CAS, 142 00 Prague, Czech Republic; Institute for Molecular Physiology, Johannes Gutenberg University, 55128 Mainz, Germany; Institute of Molecular Biology (IMB), 55128 Mainz, Germany; Institute of Molecular Biosciences, Goethe University, 60438 Frankfurt am Main, Germany; Buchmann Institute for Molecular Life Sciences (BMLS), 60438 Frankfurt am Main, Germany; Institute of Physical and Theoretical Chemistry, Goethe University, 60438 Frankfurt am Main, Germany; Institute of Molecular Biosciences, Goethe University, 60438 Frankfurt am Main, Germany; Max Planck Institute for Biophysics, 60438 Frankfurt am Main, Germany

## Abstract

Nuclear speckles (NS) and paraspeckles (PS) are adjacent yet distinct nuclear condensates that undergo stress-induced reorganization. Here, we identify a dual role for the splicing factor SRSF5 in coordinating the crosstalk between both condensates. Super-resolution imaging shows that SRSF5, while enriched in NS, also overlaps with the shell of a subset of PS. SRSF5 binds purine-rich sequences at the 5′ end of *NEAT1_2* promoting its alignment to PS shells and the formation of large PS cluster during stress. We propose that SRSF5 binding occurs transiently during PS maturation and must later be removed from *NEAT1_2* by nuclear helicases. Inhibition of this remodeling by rocaglamide A, which locks helicases onto purine-rich RNA leads to the aberrant fusion of PS and NS, which can be partially rescued by acute SRSF5 depletion. Surprisingly, while short-term SRSF5 loss impairs PS formation, prolonged depletion activates a feedback loop involving intron retention and premature polyadenylation of *TARDBP*, reduction of TDP-43 levels and *NEAT1_2* isoform switching, ultimately restoring PS clusters. Our findings reveal that SRSF5 serves both architectural and regulatory roles in PS biogenesis and that helicase-mediated remodeling is essential to maintain PS identity and function under stress. These insights uncover fundamental principles of nuclear body dynamics.

## Graphical Abstract

**Graphical Abstract:**
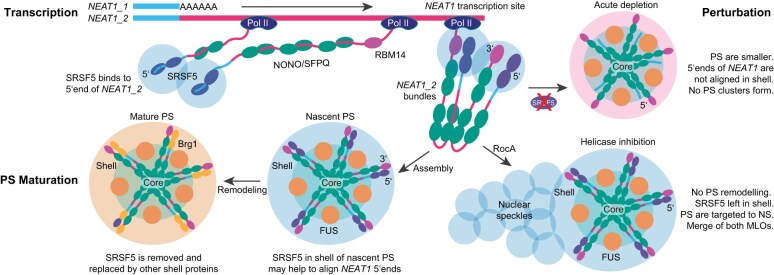

## Introduction

The nucleus of mammalian cells is compartmentalized into distinct phase-separated membrane-less organelles (MLOs), which spatially organize concurring synthesis and processing pathways and to regulate gene expression in response to stress [1–3]. Among these, paraspeckles (PS) serve as key hubs for coordinating the spatial and temporal organization of the cellular stress response [4]. PS are scaffolded by the architectural RNA *NEAT1* and contain over 80 different RNA-binding proteins (RBPs) [5–8], of which eight are essential for PS formation (DAZAP1, FUS, HNRNPH3, HNRNPK, NONO, RBM14, SFPQ, and SMARCA4) [9–11]. Most PS proteins (PSPs) contain intrinsically disordered regions (IDRs) or prion-like domains, which promote phase separation and are essential for PS assembly [12, 13]. While the precise composition of PS remains incompletely defined, it is thought to be dynamic and stress-dependent, allowing the sequestration of specific RNAs and proteins in response to different cellular stressors [14, 15].

PS assemble co-transcriptionally as PSPs bind to nascent *NEAT1* transcripts in a coordinated manner. This forces *NEAT1* to adopt a V-shaped structure and keeps monomeric *NEAT1* ribonucleoprotein particles (RNPs) in close proximity. Approximately 50 *NEAT1* RNPs then assemble into a mature phase-separated PS with an intricate core-shell structure and a fixed diameter of 360 nm [16–19]. In this architecture, the 5′ and 3′ ends of *NEAT1* are oriented towards the shell, while the middle region is hidden in the PS core. The PSP FUS acts as a separator of individual chains [18].

*NEAT1* exists in two major isoforms that share the same 5′ end region, but only the long isoform (*NEAT1_2*; 22 kb) scaffolds PS and their numbers strictly correlate. While the short *NEAT1* isoform (*NEAT1_1;* 3.7 kb) is canonically polyadenylated, the long isoform only forms when polyadenylation of *NEAT1_1* is prevented. *NEAT1_2* harbors a tRNA-like module at its 3′ end, which is cleaved off by RNase P. The resulting 3′ end is stabilized through a U–A°U triple-helix structure [8, 20]. Several RBPs are known to regulate the balance between both isoforms. For instance, HNRNPK competes with cleavage factor CPSF6 for NUDT21 binding, thereby suppressing *NEAT1_1* formation [11]. Conversely, the paraspeckle component TAR DNA-binding protein 43 (TARDBP/TDP-43) and the Integrator subunits INTS10 and INTS11 promote *NEAT1_1* expression by enhancing its polyadenylation [21, 22]. TDP-43 binds GU-rich motifs and its function is highly dosage- and context-sensitive. Its mis-regulation leads to widespread transcriptome alterations and has been implicated in neurodegenerative diseases such as amyotrophic lateral sclerosis and frontotemporal lobar degeneration. Thus, maintaining appropriate TDP-43 levels is essential for cells, but our understanding how TDP-43 levels are controlled is incomplete [23].

During stress, PS increase in size and number and form larger clusters through increased *NEAT1* transcription and enhanced polyadenylation site (PAS) read-through [1, 4]. The individual PS grow in only one dimension and form cylinder-shaped structures, consistent with a co-polymer micelle model [23, 24]. However, the precise architecture of these larger PS clusters remains poorly understood.

PS are often found in close proximity to nuclear speckles (NS)—another nuclear MLOs that functions in the sequestration of splicing factors and regulation of co-transcriptional mRNA processing [6, 25]. Of note, the RBPs that reside in PS and NS are fundamentally different. NS are enriched in RBPs with arginine and serine residues (RS domains), including serine-arginine-rich splicing factors (SRSFs), which form a dense meshwork that constitutes the NS core [26]. The NS phase border is populated by active spliceosomes and poly(A)+ RNAs [27, 28]. In contrast, inhibitory splicing factors—including heterogeneous nuclear ribonucleoproteins (hnRNPs)—are excluded from NS but are enriched in PS. Thus, the types of RBPs and their interactions provide both MLOs with very different physico-chemical properties and may help to prevent their fusion. Indeed, a recent study demonstrated that RBPs that localize to the shell of PS, e.g. SFPQ are important for the physical separation of both MLOs [29], but it is less clear how PS and NS are held in close proximity. One common element could be purine-rich RNAs, which are bound by SR proteins that localize to the NS core [30, 31], but they were also found enriched in PS distributed along the shell of PS spheroids [18].

We have recently shown that hypoxic stress partially dissolves NS and releases SRSFs, while PS increase in size and number [32]. These findings raised the possibility of a crosstalk between NS and PS during the stress response. To explore this, we focused on SRSF5, a core NS component that binds purine-rich RNAs. SRSF5 was also detected in PS by mass spectrometry (MS) [33] and shown to bind to *NEAT1* [34, 35], but its role in PS biology or NS–PS communication remains unknown.

Here, we show that SRSF5 is essential for PS assembly during stress and cellular homeostasis, acting through both direct and indirect mechanisms. SRSF5 binds co-transcriptionally to purine-rich stretches at the 5′ end of *NEAT1*, localizes to the PS shell, and promotes proper *NEAT1_2* packaging and PS cluster formation under specific stress conditions. Surprisingly, our data indicate that SRSF5 must be removed from the shell of mature PS to prevent their fusion with NS, indicating that PS are constantly remodeled via RNA helicases. Additionally, we find that SRSF5 indirectly regulates PS dynamics by controlling the expression of TDP-43 through premature polyadenylation, thereby influencing *NEAT1* isoform switching in response to prolonged SRSF5 depletion.

## Materials and methods

### Cell culture, plasmid transfection, and drug treatments

HeLa cells were cultivated under humidified condition at 5% CO_2_ and 37°C in Dulbecco's Modified Eagle Medium (DMEM; GlutaMAX Medium, supplemented with 10% (v/v) heat inactivated fetal bovine serum, and 100 μg/ml penicillin–streptomycin (all Gibco^™^, Thermo Fisher Scientific). For stress experiments, HeLa cells were grown on coverslips placed in 24-well plates and treated with different compounds, followed by fixation with 4% Paraformaldehyde (PFA; Thermo Fisher Scientific). The proteasome was inhibited with 10 μM MG132 (M7449-200UL, Sigma–Aldrich), and translation was inhibited with 5 μM rocaglamide A (SML0656-100UG, Sigma–Aldrich), 100 μM cycloheximide (CHX), or amino acid starvation using Hanks’ Balanced Salt Solution (HBSS, H8264 Merck). Oxidative stress was induced with 0.25 mM of sodium arsenite (041533-AP, Thermo Fisher Scientific), and Hypoxia Induced Factor 1α (HIF1α) was stabilized using 250 μM cobalt chloride (Sigma–Aldrich). All compounds were diluted in fresh DMEM and cells were treated for the indicated times. To induce expression of the hGRAD systems, cells were treated with 1 μg/ml doxycycline (DOX; Sigma–Aldrich, D9891). For hypoxia experiments, coverslips were placed in 6 cm plates, and control cells were either grown in normoxic or hypoxic conditions (24 h, 37°C, 0.2% O_2_, 5% CO_2_) in a Hypoxiastation H35 (Don Whitely Scientific Limited; HypoxyLab, Oxford Optronics). Transcription was inhibited using 5 μg/ml Actinomycin D (ActD).

Plasmids were confirmed by Sanger sequencing (ACGT) and extracted from bacteria with the ZR Plasmid Miniprep Classic (Zymo Research). WT HeLa cells were transfected with 0.5 μg purified plasmid DNA per well in six-well plates using the jetOPTIMUS^®^ (Polyplus-transfection). All plasmids are listed in Supplementary Table S8 and all primers in Supplementary Table S7.

### Genome editing with CRISPR–Cas9

SRSF5 KO cells were generated by a dual single guide RNA (sgRNA) approach. Two sgRNAs were chosen, which cut in the coding sequence within exon2 and exon3 of SRSF5, generating large deletions and frameshifts, subsequently leading to mRNA decay by nonsense-mediated decay. The sgRNAs were annealed from a specific crRNA and a universal tracrRNA (IDT). The CRISPR RNAs (crRNAs) were designed with the IDT design tool. Site-specific crRNAs were annealed with the universal tracerRNA to sgRNAs and diluted to a final concentration of 1 μM. The crRNA and tracrRNA were first heated to 95°C for 5 min and then slowly cooled down to RT to allow optimal sgRNA duplex annealing. Cells were transfected with pre-assembled Cas9–sgRNA RNPs using Lipofectamine CRISPRMAX (Invitrogen) and incubated for another 48 h. Cells were directly used for single-cell generation on 96-well plates. After further incubation (∼7–10 days), all potential single-cell clones were subjected to genomic screening by PCR and potential KO cells were sequenced.

The *TARDBP* locus was tagged with GFP. Primers including homology to the repair site and the top-ranking guide RNA for LbCas12a were designed using the online oligo design tool (http://pcr-tagging.com/) [36]. The repair cassette of the pMaCTag-P05 vector harboring eGFP and a puromycin resistance gene was amplified by PCR. The repair cassette was transfected together with the LBCas12a encoding helper plasmid pcDNA3.1-hAsCpf1 (TYCV) py210. Twenty-four hours later, the cells were switched to the selection medium (DMEM containing 1 μg /ml puromycin). Single clones were expanded in 96-well plates, and recombination with the repair cassette was validated using PCR and western blotting.

Sequences of gRNAs are listed in Supplementary Table S4, plasmids in Supplementary Table S8, and primers in Supplementary Table S7.

### RNA isolation, reverse transcription, and qPCR

TRIzol^™^ (Thermo Fisher Scientific) was added directly to the plates and lysates were stored at −80°C. RNA was extracted according to the manufacturer’s instructions, treated with TURBO^™^ DNase (Thermo Fisher Scientific) for 30 min at 37°C to remove genomic DNA and subsequently purified. One microgram of RNA was reverse transcribed into cDNA using SuperScript^™^, 10 mM dNTP Mix (both Thermo Fisher Scientific), and oligodT (Sigma–Aldrich). Primers for quantitative RT-PCR (qPCR) were designed using Primer-BLAST (https://www.ncbi.nlm.nih.gov/tools/primer-blast/). qPCRs were performed using cDNA (1:8 dilution) and the ORA^™^ SEE qPCR Green ROX L kit (highQu) on a PikoReal 96 machine (Thermo Fisher Scientific). Data were plotted using GraphPad Prism 8 (https://www.graphpad.com), and conditions were tested for significance using the Student’s *t*-test. Primers used are listed in Supplementary Table S7.

### Western blot and antibodies

Cells were lysed in 300 μl of NET-2 buffer [150 mM NaCl, 0.05% (v/v) NP-40, 50 mM Tris–HCl pH 7.5 supplemented with 1 × cOmplete Protease Inhibitor Cocktail (Sigma–Aldrich) and 10 mM β-phosphoglycerate (Fluka BioChemica)] or with Radio-Immuno-Precipitation Assay (RIPA) buffer [150 mM NaCl, 0.05% (v/v) NP-40, 50 mM Tris–HCl pH 7.5, 0.1% (w/v) SDS, 0.5% (w/v) sodium deoxycholate, freshly added 1 × cOmplete Protease Inhibitor Cocktail, and 10 mM β-phosphoglycerate]. NET-2 lysates were sonicated on ice for 30 s (3 pulses of 10 s; 20-s intervals) at 20% amplitude (Branson W-450 D) and cleared by centrifugation. Protein concentrations were measured using Quick Start Bradford 1 × Dye Reagent (Bio-Rad^®^) on a NanoDrop2000 (Thermo Fisher Scientific) or DC Protein-Assay (Bio-Rad^®^) for RIPA samples. About 20–40 μg protein were separated by SDS–PAGE on 4%–15% Mini-PROTEAN^®^ TGX Stain-Free^™^ Gels (Bio-Rad^®^) and transferred onto Polyvinylidene fluoride (PVDF) membranes using Trans-Blot Turbo RTA Mini LF PVDF Transfer Kit (Bio-Rad^®^). Transfer and equal loading were evaluated by activation of stain-free gels by UV light. Membranes were probed with the antibodies listed in Supplementary Table S5. Proteins were imaged using secondary antibodies coupled to a horseradish peroxidase and Amersham^™^ ECL Prime Western Blotting Detection Reagent (Cytiva) with the ChemiDoc^™^ MP Imaging System. Image quantification was performed using the ImageLab software (Bio-Rad).

### Nascent-RNA sequencing

HeLa WT and SRSF5 KO cells were incubated with 400 μM 4-thio-uridine (4sU) for 1 h to label newly transcribed RNAs. The labeling reaction was stopped by adding TRIzol^™^ reagent to cells in the culture dishes. RNA extraction and pull-down of newly transcribed RNA was performed according to [37] without fractionation of the RNA by sonification. Nascent RNA was purified using RNA Clean and Concentrate Kit (ZymoResearch). cDNA libraries for RNA-seq were prepared with universal Plus^™^ Total RNA-Seq Library Preparation Kit (Tecan) according to manufacturer’s instructions. Ribosomal RNA fragments were removed and the library was sequenced on an Illumina NovaSeq6000 instrument (two replicates per time point, 100 Mio reads, 150 bp, paired-end).

Reads were mapped to the human genome (hg38) using the STAR mapper (version 2.7.10) [38] with the following parameters: –outFilterMultimapNmax 1 –outFilterMismatchNmax 999 –outFilterMismatchNoverReadLmax 0.04 –outSAMtype BAM SortedByCoordinate. Differential gene expression was quantified using DESeq2 with default parameters [39]. Gene Ontology (GO) enrichment analysis was performed with hypeR or with the over-representation test implemented in the enrichGO function of the clusterProfiler package in R. Enrichment was tested against the union of all genes that were tested in the DESeq2 analysis. Adjusted *P-*value cutoffs were set to 0.05 and “Biological Process” categories were explored.

Splicing analysis was performed using MAJIQ (version 2.2) [40]. Specifically, we used the majiq-build function to construct a single splice graph over all replicates and conditions (WT, SRSF5 KO, T0, T2, T8, and T16). Next, majiq-deltapsi was used to quantify local splicing variations (LSVs) between WT and SRSF5 KO and T0, T2, T8, and T16 datasets. Identified LSVs from both comparisons were considered significantly regulated with a difference in junction usage (percent selected index, PSI) of deltaPSI > 5% and a changing probability >50%. Non-regulated LSVs were considered with deltaPSI < 2% and an LSV changing probability of 0%. Both regulated and non-regulated LSVs were subsequently stratified to the binary level. First, we identified the two main junctions per LSV based on the deltaPSI, retaining only those LSVs were at least 50% of the change seen in the strongest junction can be explained by the second strongest junction. LSVs were classified into the events “intron-retention,” “alternative 3′ splice site,” “alternative 5′ splice site,” and “cassette exon.” This was done by combining source and target LSV junctions for a given event, requiring both LSVs to be regulated as defined above. Cassette exon events were further sub-classified into “simple cassette exon,” “alternative first exon,” “alternative last exon,” and “complex events” using the same methodology.

### Confocal image acquisition and quantification

Images were acquired with confocal laser-scanning microscope (LSM780; ZEISS) using the Zen 2012 (black edition; 8.0.5.273; ZEISS). Fluorescence signal was detected with an Argon laser (GFP – 488 nm, Qasar 570–561 nm, and Qasar 670–647 nm). Images from the same experiment were acquired with the same settings for all conditions. Line scans were performed by drawing a straight line across the nucleus, without crossing the nucleolus. Fluorescence intensity per pixel in the line area was acquired using the “Plot Profile” tool. Images were analyzed using Fiji [41]. Pictures were cropped with the Image crop function and scale bars were added. For fluorescence quantification, the Hoechst channel was used to acquire a threshold image (“Threshold Li”), and the “Particle analyzer” plug-in from the Biovoxxel toolbox (https://imagej.net/BioVoxxel_Toolbox) was used to obtain the nuclear regions of interest (ROI). ROIs were transferred to the GFP channel and fluorescence was quantified using the “integrated density value” (mean gray value per pixel × area). Data were plotted using GraphPad Prism 8 (https://www.graphpad.com), and conditions were tested for significance using the Mann–Whitney *U*-test.

### Nanobody labeling

Unlabeled nanobody against GFP (anti-GFP single domain Antibody, #N0305-250 μg) with a single ectopic cysteine at the C terminus was purchased from NanoTag Biotechnologies. Nanobodies were self-labeled with azide-modified docking strands according to a previously published protocol [42]. In brief, 20 nmol of nanobodies in 1× phosphate buffer saline (PBS) were incubated with a final concentration of 5 mM Tris(2-carboxyethyl)phosphin-Hydrochlorid (TCEP; Sigma–Aldrich) for 2 h at 4°C to reduce the ectopic cysteine. Afterwards, the excess of TCEP was removed by exchanging the buffer to PBS pH 6.5 using Amicon spin filter (MWCO 10 kDa, Merck). The DBCO-sulfo-NHS ester linker (Jena Bioscience) was dissolved in dimethylformamide (DMF) (Sigma–Aldrich, Germany) and then diluted in 1× PBS. Linker and nanobody were mixed in a 10:1 molar ratio and incubated for 90 min at 4°C gently shaking. The unbound linker was removed using Amicon spin filter (MWCO 10 kDa). Azide-modified P1 docking strand (Supplementary Table S3) and linker–nanobody conjugate were incubated in a 10:1 molar ratio overnight at 4°C while slightly shaking. The next day, unbound DNA was removed using an Amicon spin filter (MWCO 10 kDa). The docking strand-labeled nanobody was stored at 4°C.

### RNA fluorescence *in situ* hybridization (FISH) and immunofluorescence (IF)

For fluorescence *in situ* hybridization (FISH) and immunofluorescence (IF) experiments, 12-mm coverslips were placed inside 10-cm plates used for the experiments. After removing the medium and washing the cells with 1× PBS, the coverslips were transferred to 24-well plates. Cells were fixed with 4% PFA in PBS for 15 min, washed with PBS, and permeabilized with 70% ethanol for 1 h. FISH was performed using Stellaris probes and buffers (LG Biosearch Technologies) following the manufacturer’s protocol. Coverslips were washed with Stellaris Wash Buffer A followed by incubation in Stellaris Hybridization buffer containing the FISH probes and the primary antibodies, placed in a humidified chamber and hybridized for 16 to 20 h at 37°C protected from light. Mouse α-SRRM2 antibody (PA5-59559, 1:70 dilution in Stellaris Hybridization buffer) was used to detect NS. To visualize stress granules (SGs), mouse α-G3BP1 antibody (ab56574, 2 μg/ml final concentration in Stellaris Hybridization buffer) was used as a marker. After hybridization, the coverslips were incubated with Stellaris Wash Buffer A containing the secondary antibodies in a 1:500 dilution for 30 min at 37°C. DNA was stained with Hoechst 34580 (Sigma–Aldrich) at a final concentration of 0.25 μg/ml in Wash Buffer A for 30 min at 37°C. The coverslips were washed with Wash Buffer B, dried for 15 min and mounted onto glass slides using ProLong Diamond Antifade Mountant (Invitrogen). FISH probes used are listed in Supplementary Table S2 and antibodies in Supplementary Table S5.

For super-resolved IF, fixed cells were blocked using immunofluorescence staining (IF) buffer [3% (w/v) bovine serum albumin (BSA), 0.1% (v/v) Triton-X 100 in 1× PBS] for 30 min and in the following incubated with the self-labeled anti-GFP nanobodies at a concentration of ∼50 nM in IF buffer for 1.5 h at room temperature while shaking. Afterwards, the samples were washed three times with 1× PBS and then fixed with 4% (v/v) formaldehyde for 10 min at room temperature and washed three times with 1× PBS. Gold beads (Gold nanoparticles, 100 nm diameter, Product #A11-100-NPC-DIH-1–25, NanoPartz, USA) as fiducial markers were diluted 1:5 in 1× PBS and incubated for 10 min.

### dstorm/DNA-paint imaging

*d*STORM and DNA-PAINT imaging of HeLa cells were carried out on a home-built widefield setup based on a Nikon Eclipse Ti microscope [43]. The excitation light was generated by a DPSS laser at 640 nm (LPX-640L-500-CSB-PPA, Oxxius S.A) and a laser diode at 488 nm (LBX-488–200-CSB-PPA, Oxxius S.A) with the required excitation power controlled by an acousto-optic tunable filter (AOTFnC-400.650-TN, AA Opto Electronic). To clean the beam-profile the laser was coupled by a fibre collimator (60FC-4-M6.2-33, Schäfter & Kirchhoff GmbH) into a polarization maintaining single-mode optical fiber (PMC-E-400RGB, Schäfter & Kirchhoff GmbH) and subsequently recollimated to a FWHM diameter of 6 mm (60FC-T-4-M50L-01, Schäfter & Kirchhoff GmbH). The collinear beam was then directed through two telescope lenses (AC255-030-A-ML and AC508-150-A-ML, Thorlabs GmbH), which focused the beam onto the back focal plane of the objective (CFI Apochromat TIRF 100XC Oil, NA 1.49, Nikon). A mirror mounted on a motorized translation stage (MTS50-Z8, Thorlabs GmbH) was used to vary the illumination angle between widefield, HILO, or TIRF. The excitation light was coupled into the microscope by means of a dielectric beamsplitter (zt405/488/561/640rpc, AHF Analysentechnik), which also transmitted the emission light into the detection beam path. The axial focus was maintained using an autofocus system (Ti-PFS, Nikon) and the lateral position was adjusted using a motorised stage (Ti-S-ER, Nikon) combined with a piezo stage (Nano-Drive, MadCityLabs). After spectral filtering with a bandpass filter (700/75 ET, Chroma Technology Corporation), the emission light was projected onto an Andor Ixon Ultra EMCCD camera (DU-897U-CS0, Andor). *NEAT1* samples were excited with a laser intensity of 135 W/cm² and SRSF5 with 215 W/cm² in HILO illumination.

*d*STORM imaging of *NEAT1* was performed in *d*STORM imaging buffer [1× PBS, 100 mM β-mercaptoethylamine, 50 U/ml glucose oxidase, 5000 U/ml catalase, 0.2 mM Tris (2-carboxylethyl) phosphine hydrochloride, 20% (w/v) glucose, pH 8.5]. Before the start of the *d*STORM measurement, the sample was illuminated for 7.5 min to transform Cy5 into the dark state [44]. A preamplifier gain of 3 and an EM-gain of 200 were used to record 35 000 frames with an integration time of 30 ms. For the combined *d*STORM and DNA-PAINT two-color measurements, following *d*STORM imaging, cells were washed with 1× PBS and then irradiated with 640 and 488 nm laser for 30 min to photobleach all remaining Cy5 fluorophores. DNA-PAINT measurement of SRSF5 or NEAT1 was performed in DNA-PAINT imaging buffer (1× PBS, 0.5 M NaCl) containing the imager strand (Supplementary Table S3) at 1 nM concentration. A total of 30 000 frames with an integration time of 80 ms were recorded.

### dstorm/DNA-paint data post processing

*d*STORM and DNA-PAINT data were post processed with the Picasso software (v0.6.0) [45]. Localization of single molecule signals was performed using Picasso Localise module. The following parameters were used: baseline 82.3 photons, sensitivity 5.14, quantum efficiency 0.95, pixel size 158 nm, and a min net gradient of 8000 for *NEAT1* and 10 000 for SRSF5. Reconstruction of the super-resolution images was performed using Picasso Render module. The images were drift corrected with redundant cross correlation (RCC) and stacks of 2000–4000 frames. Two color images were aligned using gold beads as fiducial markers (Gold nanoparticles, 100 nm diameter, Product #A11-100-NPC-DIH-1-25, NanoPartz). Localizations from *NEAT1* were filtered for the ellipticity (0–0.15) and the width of the point spread function (0.8–1.15 px) and linked with 4x NeNA and 2 transient dark frames. Localizations from SRSF5 were filtered for the ellipticity (0–0.2) and the width of the point spread function (0.7–1.3 px) and linked with 4x NeNA and six transient dark frames. Images were exported with a 10 nm pixel size.

### dstorm/DNA-paint data analysis

For the threshold-based segmentation of the paraspeckle signal, custom-written Fiji macros were used. Therefore, the super-resolved images were duplicated, smoothed with a Gaussian blur of 10 nm, and then converted into a binary mask. The necessary threshold was first tested manually and then applied to all measurements. The objects were then counted and measured automatically with the “Particle analyzer” plug-in from the Biovoxxel toolbox (https://imagej.net/BioVoxxel_Toolbox). To quantify the frequency and size of individual PS, the segmented objects were filtered by the following parameters: size (>0.025 μm²) and roundness (>0.8). Roundness is defined as:


\begin{eqnarray*}
{\rm Round} = \ \frac{{4 \cdot {\rm Area}}}{{\pi \cdot {\rm major}\_{\rm axi}{{{\rm s}}^2}}}
\end{eqnarray*}

Size (>0.2 μm²) was filtered to quantify paraspeckle clusters. The categorization into “small” and “large” PS was based on a fixed threshold radius of 140 nm. Two-sample *t*- and Mann–Whitney *U*-tests were performed using GraphPad PRISM (GraphPad Software).

### STED imaging

Two-color STED imaging of *NEAT1* was performed using an Abberior Expert Line microscope (Abberior Instruments) equipped with an Olympus IX83 stand (Olympus Deutschland GmbH) and a UPLXAPO 60× NA 1.42 oil immersion objective (Olympus Deutschland GmbH). For image acquisition, the sample was excited with a 561- and a 640-nm excitation laser (1.5 and 16 μW, respectively, at the back focal plane) and depleted with a pulsed 775-nm laser (506 and 17 mW at the back focal plane) using a 2D donut point broadening function and a delay of 750 ps, and a detection width of 8 ns. Fluorescence signals were acquired in the spectral regions from 571 to 630 nm and from 650 to 760 nm using two avalanche photodiode (APD) detectors. Images were acquired with a pinhole aperture of 0.81 AU, a line accumulation of 60, a pixel dwell time of 5 μs, and a pixel size of 20 × 20 nm². Measurements were acquired in line sequential mode. For STED-PAINT of *NEAT1*, imager strands (Supplementary Table S2) were diluted to a concentration of 100 nM in DNA-PAINT imaging buffer (1× PBS and 0.5 M NaCl).

### STED data quantification

The analysis of the two-colored PS in the STED images was performed with the help of a self-written MATLAB (MathWorks) script. In summary, the round signals of the 5′ probes were segmented using the Hough transformation with a sensitivity of 0.85 and then matched to the corresponding 3′ signals. The segmented signals were then fitted using a ring function:


\begin{eqnarray*}
{{C}_1} &+& {{C}_2} \cdot \exp ( - ({{((x - a)/{{u}_1})}^2} + {{((y - b)/{{v}_1})}^2}))\nonumber\\ &&+ {{C}_3} \cdot \exp ( - ({{((x - a)/{{u}_2})}^2}\nonumber\\ &&+ {{((y - b)/{{u}_2})}^2} + {{((y - b)/({{v}_1} \cdot {{u}_2}/{{u}_1}))}^2}))
\end{eqnarray*}

The distance between the center of the ring and the outer FWHM was taken as the radius of the ring-shaped 5′ or 3′ signal. The PS were categorized based on their 5′ radius, setting a cut-off radius of 280 nm for PS measured in untreated cells. PS with a smaller radius were assigned as “small,” and those with a larger radius as “large.” Using this cut-off radius, the 5′/3′ ratio was calculated for PS in untreated cells and in cells treated for 6 h with DOX.

To calculate the PCC between *NEAT1* RNA and SRSF5 signal confocal and STED images were first cropped to the size of nuclei. PCC was computed using the standard implementation provided by the SciPy statistical library (Python, scipy.stats). The “Randomized” image pair was generated by a horizontal mirroring of the *NEAT1* channel, i.e. along the vertical image axis.

To manually count PS that overlapped with SRSF5 clusters, images were cropped to the size of nuclei. Both channels were segmented using a threshold-based approach and counted. The “Randomized” image pair was generated by a horizontal mirroring of the *NEAT1* channel, i.e. along the vertical image axis.

Averaged radial intensity plots of SRSF5 signal around PS were generated in two steps. At first PS centers were identified using a threshold-based segmentation of the *NEAT1* RNA signal. In a second step normalized integrated SRSF5 intensities around concentric circles as a function of distance from the obtained PS center coordinates were plotted using the Radial Profile Plot plugin in Fiji [41]. To obtain averaged plots all intensities belonging to a specific distance were averaged. The “Randomized” image pair was generated by a horizontal mirroring of the *NEAT1* channel, i.e. along the vertical image axis.

## Results

### SRSF5 localizes to nuclear speckles and paraspeckles and binds to the 5′ end of NEAT1

**Figure 1.**
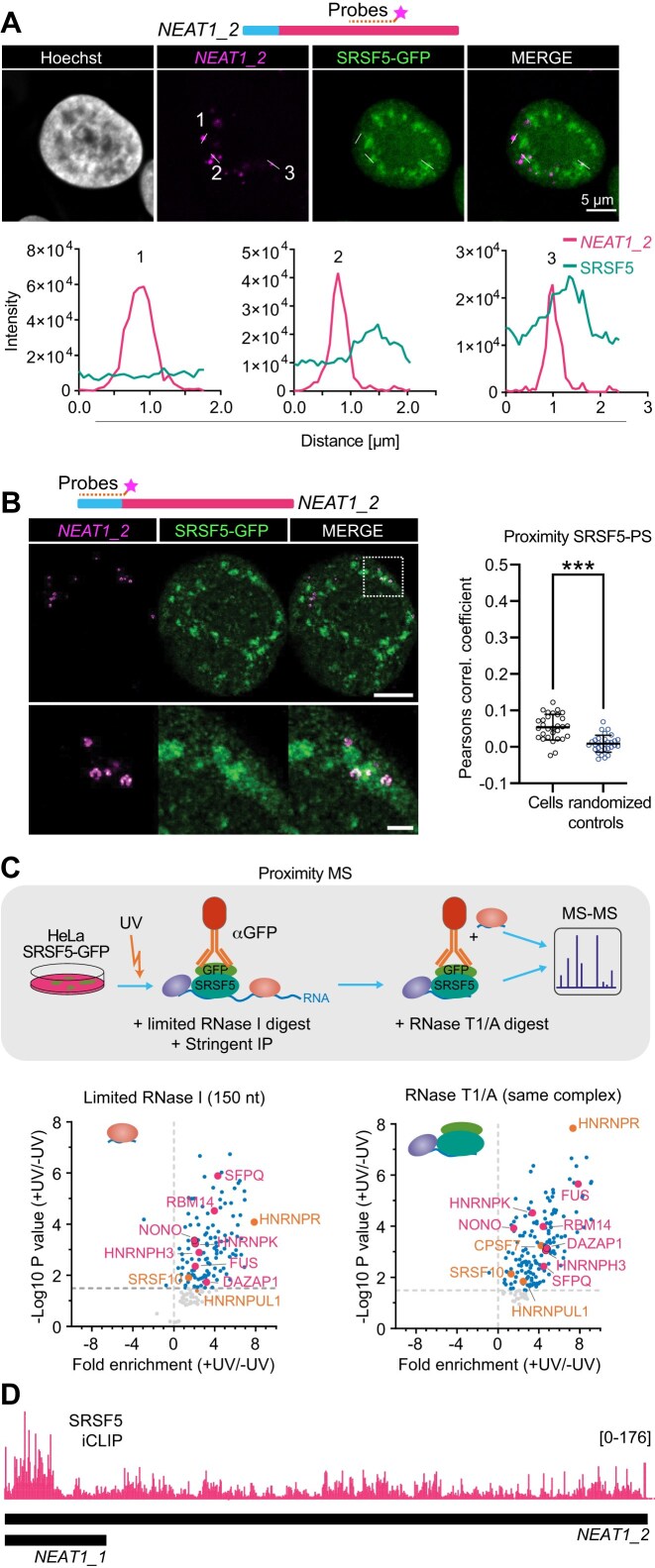
SRSF5 localizes to NS and PS and binds to the 5′ end of *NEAT1*. (**A**) Top: Confocal images of HeLa SRSF5-GFP cells showing that SRSF5 signal overlaps with PS labeled by RNA-FISH targeting the middle region of *NEAT1_2*. Nuclei were stained with Hoechst. Bottom: Example line scans show (i) no co-localization, (ii) close proximity, and (iii) partial overlap of SRSF5-GFP and *NEAT1_2*. (**B**) Left: STED microscopy and quantification of *n* = 30 cells reveal that 18.4% of PS co-localize with SRSF5 speckles. Right: The PCC shows that the overlap is significantly higher than for randomized controls. (**C**) Top: Scheme of the proximity MS workflow that identified all proteins binding in close proximity to SRSF5 to the same short fragment of RNA [50]. Proteins that bind to SRSF5 via protein–protein interactions, usually occurring within NS, are not enriched in the +UV samples, and thus are not captured with this assay. Bottom: RNase-sensitive (left) and partial RNase-resistant (right) SRSF5 pull-downs. Essential PSPs = pink; important PSPs = orange. (**D**) Browser shot showing the preferential binding of SRSF5 at the 5′ end of the *NEAT1_2* transcript determined by iCLIP2 [46]. Statistics: two-sided *T*-test; ***P < 0.001..

A recent study suggested that the NS-resident protein SRSF5 may also localize to PS [33]. To confirm this dual localization, we examined the subnuclear distribution of endogenously tagged SRSF5-GFP in HeLa cells [46]. NS were visualized by IF staining for the NS marker SRRM2, while PS were detected by RNA fluorescence *in situ* hybridization (RNA-FISH) using probes targeting the middle region of *NEAT1_2*. Confocal microscopy revealed that SRSF5 was enriched in NS but also distributed throughout the nucleoplasm (NP) with a nuclear speckle-to-NP signal ratio of 2.4:1 (Supplementary Fig. S1A). Line scan analyses identified three spatial patterns: (i) PS signals were distant from both SRSF5 and NS; (ii) SRSF5 signals were in close proximity to PS; and (iii) SRSF5 signals overlapped with PS signals (Fig. 1A).

To quantitatively assess the overlap of NS and PS, we employed super-resolution stimulated emission depletion (STED) microscopy. The 5′ end of *NEAT1_2* was labeled with RNA-FISH probes that were extended with a short DNA sequence (see “Materials and methods” section), and 30 cells were imaged [43, 47–49] (Fig. 1B). The colocalization between PS and SRSF5 signal was compared to randomized controls (Supplementary Fig. S1B) by calculating the PCC. The PCC was slightly but significantly higher than for the randomized controls. In addition, we counted the overlap of PS with SRSF5 clusters, excluding diffuse nucleoplasmic SRSF5 signal. This overlap counting revealed that 18.4% of PS (7 out of 38) showed an overlap with SRSF5 speckles—significantly above the 0% overlap seen in the randomized controls (Fig. 1B). These results suggest that a subset of SRSF5 associates with PS under steady-state conditions.

RBPs that are part of PS bind in close proximity to the scaffolding RNA *NEAT1_2*. To determine whether SRSF5 is among those RBPs, we re-analyzed our previously published SRSF5 proximity MS dataset [50]. This method combines UV crosslinking, titrated RNase digestion, and SRSF5 pulldown to identify proteins that bind in close spatial proximity on the same RNA fragment. Enrichment relative to a non-UV-treated control identified all proteins that bind in close proximity to SRSF5 on the same short RNA fragment. Consistent with its association with PS, many known paraspeckle proteins (PSPs)—including FUS, HNRNPK, NONO, RBM14, and SFPQ—were strongly enriched in UV-crosslinked SRSF5 pulldowns after limited RNase I digestion (generating ∼150 nt RNA fragments) as well as complete RNase T1/A digestion (which preserves only tightly bound complexes) (Fig. 1C and Supplementary Table S1).

To determine whether these interactions are mediated through *NEAT1*, we inspected an SRSF5 iCLIP2 dataset generated with the same cell line [46]. SRSF5 binds to *NEAT1_2* along its entire length but showed a marked preference for its 5′ end (Fig. 1D). The SRSF5 binding profile was distinct to binding profiles of other PSPs, including NONO, hnRNPK, PSPC1, or TDP-43, but resembled that of the PSP TAF15 [51], which was notably absent in the SRSF5 proximity MS dataset (Supplementary Fig. S1C and Supplementary Table S1). Together, these results support the idea that a subset of NS-resident SRSF5 localizes to PS where it binds preferentially to the 5′ end of *NEAT1_2*. They further suggest that SRSF5 might occupy the shell region of some PS spheres in a mutually exclusive manner with other PSPs.

### Acute depletion of SRSF5 decreases paraspeckle number and size

**Figure 2.**
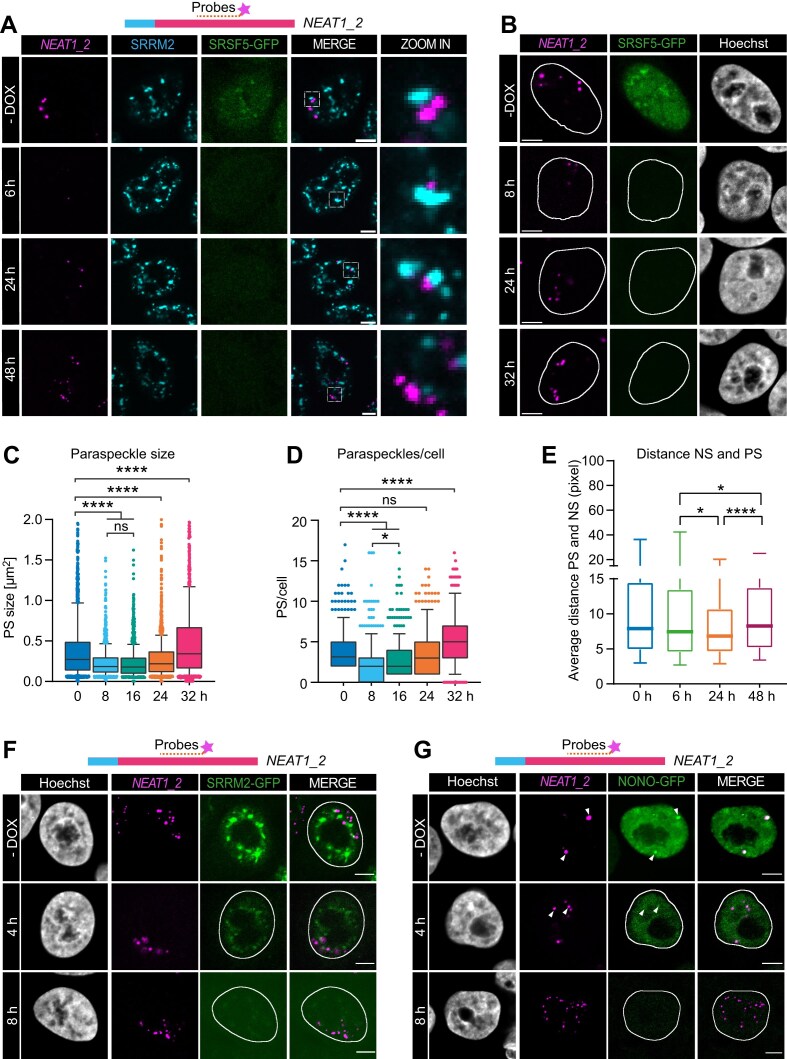
Acute depletion of SRSF5 decreases paraspeckle number and size. (**A**) Confocal images showing a time course of SRSF5-GFP depletion (6, 24, and 48 h) using hGRAD. SRRM2 was immunostained with an anti-SRRM2 antibody. (**B**) Time course of SRSF5-GFP depletion (8, 24, and 32 h) using hGRAD. (**C**) Quantification of PS size from *n* = 319–375 cells and (**D**) PS number per cell from *n* = 319–375 cells. (**E**) Quantifications of the average distances between PS and NS from *n* = 549–977 PS from the time course of SRSF5 depletion (6, 24, and 48 h). (**F** and **G**) Time course of SRRM2-GFP (E) and NONO-GFP (F) depletion (-DOX, 4 and 8 h) using hGRAD. All: Nuclei were stained with Hoechst. PS were labeled by targeting the middle region of *NEAT1_2*; scale bars: 5 μm. Statistics: two-tailed Mann–Whitney test; *P < 0.05 and ****P < 0.0001.

To investigate whether SRSF5 plays a functional role in PS biology, we utilized hGRAD, our recently developed DOX-inducible system for the rapid degradation of nuclear RBPs tagged with GFP [46]. HeLa hGRAD cells expressing endogenously GFP-tagged SRSF5 were treated with DOX (1 μg/ml), resulting in hGRAD-mediated degradation of SRSF5 within 2 h (Supplementary Fig. S2A). Strikingly, acute depletion of SRSF5 (8 h) had no significant impact on NS (Fig. 2A and Supplementary Fig. S2B) but led to a dramatic reduction in both the number and size of PS. In fact, PS virtually disappeared from the nuclei (Fig. 2A–D). In addition, we observed a small but statistically significant decrease in the distance between NS and the few remaining PS (Fig. 2A and E). To ensure that these effects were specific to SRSF5, we performed control experiments. Depletion of the NS core protein SRRM2 using hGRAD had no effect on PS number or morphology (Fig. 2F and Supplementary S2A), indicating that the observed PS disappearance was not due to hGRAD activation or to the general perturbation of NS core components. As expected, acute depletion of the PS core protein NONO also led to PS disassembly. However, in this case, small, evenly distributed *NEAT1_2* foci remained detectable throughout the nucleus in contrast to the near-complete loss seen upon SRSF5 depletion (Fig. 2G and Supplementary S2A). Importantly, in cells with transient re-expression of SRSF5-mCherry, PS numbers remained stable despite DOX induction and SRSF5 loss (Supplementary Fig. S2C), suggesting that the observed effect is specific to SRSF5 depletion. Together, these findings suggest that SRSF5 is required for the assembly and/or stability of PS and may influence the proximity or communication between these two MLOs.

### SRSF5 promotes correct packaging of NEAT1 and assembly of paraspeckle clusters

**Figure 3.**
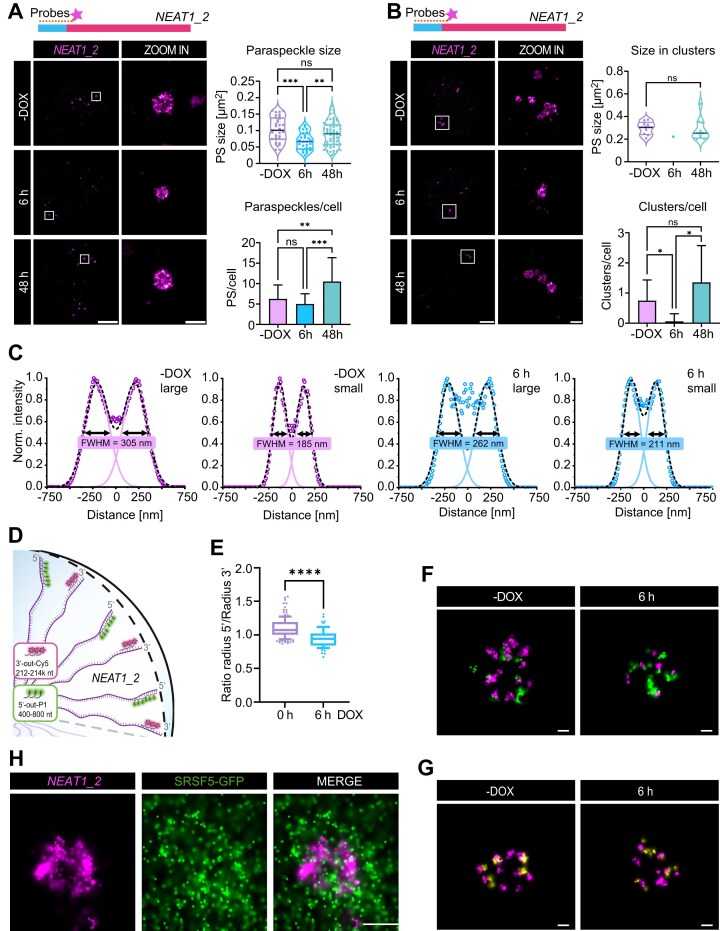
SRSF5 promotes correct packaging of *NEAT1* and assembly of paraspeckle clusters. (**A** and**C**) Direct Stochastic Optical Reconstruction Microscopy (*d*STORM) imaging PS after 6 and 48 h SRSF5-GFP depletion using hGRAD. PS were labeled by RNA-FISH using probes targeting the 5′ end of *NEAT1_2*. (A) Quantification of PS size and PS number in PS spheres from *n* = 8 cells; scale bars: 5 μm and 500 nm. (**B**) Quantification of PS that are part of larger clusters and number of clusters per cell from *n* = 7–9 cells; scale bars: 5 μm and 500 nm. (**C**) Distance distribution of normalized 5′ end signal from the center of large and small PS comparing control (0 h) and SRSF5-depleted (6 h, 1 μg/ml DOX) cells; FWHM, full width at half maximum; *n* = 8 PS. (**D**) Exemplary labeling scheme for two-color super-resolution imaging using fluorophore- or DNA-labeled RNA-FISH probes to target the 5′ and 3′ ends of *NEAT1_2*. Positions of where the probes bind to the *NEAT1_2* RNA are indicated. (**E**) Quantification of the ratio of 5′ and 3′ radii from STED imaging data. (**F**) Exemplary *d*STORM/DNA-PAINT image of PS spheres at 0 and 6 h SRSF5 depletion using RNA-FISH probes targeting the 3′ and 5′ ends of *NEAT1_2*. (**G**) Exemplary dSTORM/DNA-PAINT images of PS spheres at 0 and at 6 h SRSF5 depletion (left) labeled at two neighboring regions at the 3′ end of *NEAT1_2* using RNA-FISH probes; scale bars: 100 nm. (**H**) Exemplary *d*STORM/DNA-PAINT image targeting the 5′ end of *NEAT1_2* using RNA-FISH probes and DNA-labeled GFP nanobodies targeting SRSF5-GFP; scale bars: 200 nm. Statistics: two-sided T-test; *P < 0.05, **P < 0.01, ***P < 0.001, ****P < 0.0001.

To further dissect the role of SRSF5 in PS organization, we used DNA points accumulation for imaging in nanoscale topography (DNA-PAINT) [43, 45] and direct stochastic optical reconstruction microscopy (dSTORM) [52] to visualize individual PS in HeLa cells with superior resolution. After 6 h of acute SRSF5 depletion, RNA-FISH targeting the 5′ end of *NEAT1_2* revealed that PS were still present, but significantly smaller in diameter (317 nm) compared to untreated cells (362 nm; Fig. 3A). Moreover, only isolated PS spheres were detectable and no larger PS clusters (Fig. 3B), together explaining why PS escaped detection by standard confocal microscopy.

Under steady-state conditions, PS are thought to have a constant diameter of ∼360 nm due to the fixed length and defined packaging of *NEAT1_2*, with its 5′ and 3′ ends localizing to the shell and the middle region to the core of PS spheres [17, 29]. We confirmed this diameter in untreated HeLa cells. Thus, the reduced size of PS upon SRSF5 depletion suggest that *NEAT1_2* RNAs are either shortened or differently packaged (Fig. 3A). To test this, we analyzed the distribution of *NEAT1_2* 5′ end signal in control (-DOX) and SRSF5-depleted cells (6 h) using full width at half maximum (FWHM) measurements (Fig. 3C, see “Materials and methods” section). While large PS had a FWHM of ∼305 nm in control conditions, this decreased to 262 nm upon SRSF5 loss, suggesting that PS become smaller or that the 5′ end is more compacted. In contrast, small particles (present at low frequency in control cells) did not show such a change (FWHM: 185–211 nm). Strikingly, in control cells, 5′ end signals were excluded from the PS center, as expected, but after 6 h of SRSF5 depletion, both, large and small PS showed 5′ end *NEAT1_2* signals within the center of PS. This suggests that the shell localization of *NEAT1_2* is impaired upon SRSF5 depletion and *NEAT1_2* 5′ ends mis-localize to the PS core, which might disrupt shell integrity (Fig. 3C).

To validate this alternative packaging in small PS, we performed dual-color STED imaging of the 5′ and 3′ ends of *NEAT1_2* [43, 47–49] (see “Materials and methods” section; Fig. 3D and Supplementary S2D), which allowed for higher throughput imaging and a more robust statistical analysis. We first used a single-particle analysis and grouped PS into small and large particles based on their 5′ radius (see “Materials and methods” section). We next calculated the 5′/3′ ratio to assess the spatial organization. This analysis revealed a statistically significant reduction in the 5′/3′ ratio of small PS in SRSF5-depleted cells (6 h) compared to large PS in control (-DOX) cells, consistent with a more internalized 5′ end with respect to the 3′ label (Fig. 3E).

Next, we conducted *d*STORM/DNA-PAINT experiments of dual-labeled PS to validate this finding, and although a statistical analysis was impaired by the small throughput, single representative images confirmed that PS were smaller and the 5′ end was more internalized compared to control PS (Fig. 3F). In contrast, control experiments using two neighboring 3′ probes showed no shift in localization, confirming that the internalization is specific for the 5′ end (Fig. 3G).

To directly visualize SRSF5 localization relative to normal PS we performed *d*STORM/DNA-PAINT imaging of SRSF5-GFP and *NEAT1_2* 5′ ends. These experiments showed that PS spheres are surrounded by SRSF5, which is also enriched between PS spheres (Fig. 3H and Supplementary S2E). Along with the preference of SRSF5 for the 5′ end of *NEAT1_2*, these findings suggest that SRSF5 is required for proper shell localization and packaging of *NEAT1_2* as suggested for other shell proteins [9]. In its absence *NEAT1_2* packaging is perturbed, resulting in smaller, structurally altered PS that that fail to form larger clusters.

### Long-term depletion or SRSF5 KO triggers a PS over-compensation mechanism

While our acute depletion experiments suggested that SRSF5 is important for the correct assembly of PS, extended SRSF5 depletion (32–48 h) revealed a surprising recovery of PS. After 32 h of SRSF5 depletion, PS number, size, and their distance to NS increased relative to control cells (-DOX; Fig. 2A–E). This unexpected recovery was confirmed by *d*STORM super-resolution imaging using RNA-FISH probes targeting the 5′ end of *NEAT1_2*. After 48 h of SRSF5 depletion, PS became more numerous than in untreated cells, they recovered their original diameter (360 nm) and frequently assembled into large PS clusters (Fig. 3A and B).

**Figure 4.**
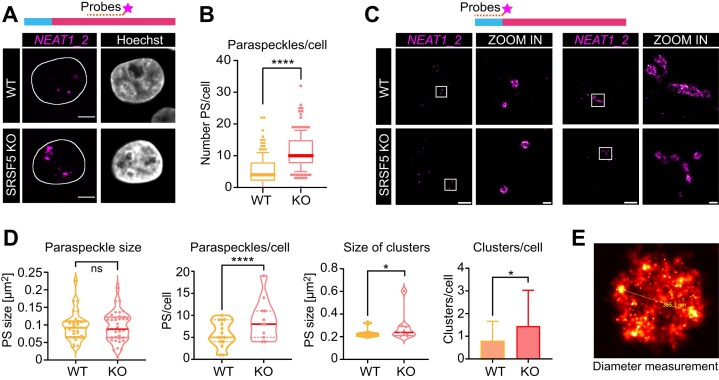
SRSF5 KO triggers a PS over-compensation mechanism. (**A**) Confocal imaging comparing PS number in HeLa WT and SRSF5 KO cells. PS were labeled by RNA-FISH using probes hybridizing to the middle region of *NEAT1_2*; scale bars: 5 μm. (**B**) Quantification of PS number per cell from *n* = 300 cells. (**C**) *d*STORM imaging of WT and SRSF5 KO cells. PS were labeled by RNA-FISH using probes hybridizing to the 5′ end of *NEAT1_2*. Left: Examples of individual PS spheres, Right: Examples of larger PS clusters; scale bars: 5 μm and 500 nm. (**D**) Quantification of the size of PS spheres, the number of PS spheres per cell, the size of PS clusters, and the number of PS clusters per cell from *n* = 9 cells. (**E**) Example micrograph from one PS sphere with a diameter of 365 nm using probes hybridizing to the 5′ end of *NEAT1_2*. Statistics: Two-sided *T*-test; *P < 0.05, ****P < 0.0001.

To investigate this apparent recovery, we generated a stable SRSF5 knockout (KO) HeLa cell line using CRISPR–Cas9 (Supplementary Fig. S3A–C). Both confocal and *d*STORM imaging revealed a phenotype similar to that of long-term depletion via hGRAD: KO cells exhibited significantly more PS than wild-type (WT) cells (Fig. 4A–D), along with an increased number of PS clusters and elongated PS structures. Notably, PS spheres in KO cells also regained a normal diameter of 360 nm (Fig. 4D and E). Additionally, the distance between PS and NS was significantly increased compared to WT cells (Supplementary Fig. S3D). Taken together these results indicate that the effects of acute versus prolonged SRSF5 depletion differ markedly. While short-term SRSF5 loss disrupts PS formation and packaging, longer depletion periods appear to trigger a compensatory mechanism that restores PS number, size, and clustering.

### Acute SRSF5 depletion reduces NEAT1 transcript output

**Figure 5.**
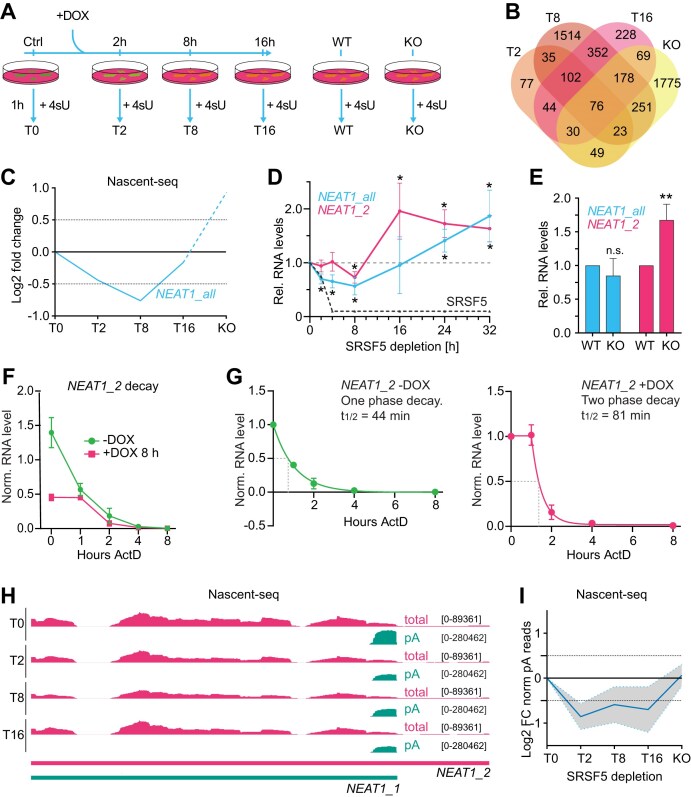
Acute SRSF5 depletion reduces *NEAT1* transcript output. (**A**) Scheme of the workflow combining a time course of hGRAD degradation as well as WT and SRSF5 KO with Nascent-seq. Cells were induced with DOX (1 μg/ml) for the indicated times, then treated for 1 h with 4sU (400 μM) to label all new transcripts followed by total RNA extraction, biotinylation, purification, and conversion into a cDNA library for deep sequencing. (**B**) Venn diagram of differentially expressed transcripts at T2, T8, and T16 compared to T0 and KO compared to WT. (**C**) *NEAT1* shows dynamic transcript changes upon SRSF5 depletion. (**D**) RT-qPCR confirms the dynamic *NEAT1* regulation after SRSF5 depletion with total RNA. (**E**) RT-qPCR confirms the over-shooting of *NEAT1* levels in SRSF5 KO cells. RNA levels were normalized to U6 snRNA. Graphs show mean and SD of *n*= 3 independent experiments. (**F**) HeLa WT cells were treated with DMSO or DOX (1 μg/ml, 8 h) to degrade SRSF5. Transcription was subsequently blocked by adding ActD (5 μg/ml) and samples were taken after 1, 2, 4, and 8 h. *NEAT1_2* transcript levels were quantified by RT-qPCR and normalized to *U6* RNA. *n* = 3 biological replicates. Error bars are SEM. (**G**) Transcript decay curves for *NEAT1_2* with 8 h DOX (right panel) or without DOX (left panel). Best fits were modeled by one-phase decay (right panel) or plateau followed by one-phase decay (left panel). (**H**) Genome browser view showing total and polyA Nascent-seq reads from the different time points of SRSF5 depletion mapping around the poly(A) site of *NEAT1_1*. The *NEAT1_2* transcript is truncated for better visibility. (**I**) Log2 fold change in the number of *NEAT1_1* reads with polyA tails quantified from the Nascent-seq data and normalized to T0. Statistics: Two-sided T-test; * *P* < 0.05, ** *P* < 0.01.

To investigate the mechanism underlying the initial loss and later over compensation of PS upon SRSF5 depletion, we analyzed changes in the nascent transcriptome using Nascent-seq. This method combines different timepoints of SRSF5 depletion using hGRAD with sequencing of newly transcribed RNAs [46, 53]. In addition to acute depletion, we generated a Nascent-seq dataset from HeLa SRSF5 KO cells to study the effect of long-term depletion (Fig. 5A).

HeLa hGRAD-SRSF5-GFP cells were treated with DOX and after different time points of SRSF5 depletion (2 h, T2; 8 h, T8; 16 h, T16) nascent transcription was labeled by incubating cells with 400 μM 4-thiouridine (4sU) for 1 h. For comparison, the same labeling protocol was applied to SRSF5 KO and WT cells. 4-SU-labeled RNAs were biotinylated, purified, and sequenced allowing the analysis of dynamic gene expression and RNA processing changes upon SRSF5 loss. Untreated cells (T0) and WT cells served as controls.

Differential gene expression analysis using DESeq2 [39] revealed very rapid and dynamic changes in hundreds of nascent transcripts following SRSF5 depletion (Fig. 5B). Among these, *NEAT1* displayed a particularly dynamic response—it was down-regulated at T2 and T8, partially recovered at T16 and strongly increased (∼2-fold) in SRSF5 KO cells (Fig. 5C). This dynamic response was also seen by RT-qPCR using total RNA, showing a similar transient downregulation (2–8 h), recovery and later 2-fold overexpression of *NEAT1* (*NEAT1_all*) at 32 h and in SRSF5 KO cells (Fig. 5D and E). These data mirror the changes in PS numbers and structure observed over time (Figs 2D and 3A–B). To confirm this relationship, we induced *NEAT1* transcription in WT cells for 6 and 12 h using CoCl_2_, which stabilizes the *NEAT1* transcription factor HIF2A [54]. This treatment increased the levels of *NEAT1_2* progressively (1.5- and 2.8-fold), with a comparable increase in PS numbers (1.1 and 2.5-fold) and size (1.5- and 2.3-fold) (Supplementary Fig. S4A). This indicates that *NEAT1* transcriptional output and number of PS are directly related [5].

The transient loss of *NEAT1* after acute SRSF5 depletion suggests either impaired transcription or destabilization of *NEAT1* transcripts. To distinguish between these possibilities, we used ActD to inhibit transcription and measured *NEAT1* decay over time [55]. To improve the extractability of *NEAT1_2* RNAs, we applied a heat treatment (55°C, 10 min), which strips-off bound proteins [56]. As suggested by the Nascent-seq data, *NEAT1_2* levels were drastically reduced (3.1-fold) when SRSF5 was depleted (8 h DOX; 0 h ActD) (Fig. 5F). But surprisingly, despite its lower levels, residual *NEAT1_2* decayed slower after acute depletion of SRSF5 (8 h) exhibiting a two-phasic decay curve and half-lives of 80 min compared to 44 min in control cells (-DOX) (Fig. 5G). These values are similar to *NEAT1_2* half-lives measured in previous studies in other cell types (N2A cells, 63 min [57]; K562 cells, 84 min [58]). Our data indicate that reduced stability does not account for the loss of *NEAT1* RNAs after acute SRSF5 depletion. They suggest that rather *NEAT1* transcription or processing is impaired. Since *NEAT1* is a very unstable RNA, reduced *NEAT1* transcription and high turnover likely contribute to the transient disappearance of *NEAT1* and PS when SRSF5 is absent.

### A TDP-43–mediated isoform switch promotes NEAT1_2 upregulation and PS over-compensation

To investigate the compensatory mechanism that restores *NEAT1_2* levels after prolonged SRSF5 depletion times or complete KO, we examined the relative abundance of the two *NEAT1* isoforms over time. The long *NEAT1_2* isoform, which scaffolds PS, is produced by read-through transcription beyond the polyadenylation signal (PAS) of the short *NEAT1_1* isoform [11, 59]. By counting only reads containing non-templated adenines (pA) that map to the *NEAT1* locus, we found that the short polyadenylated *NEAT1_1* isoform progressively decreased after SRSF5 depletion (Fig. 5H and I). In contrast, *NEAT1_2* levels increased very early and remained elevated in SRSF5 KO cells (Fig. 5D). These results suggest that PS overcompensation might be driven, at least in part, by an early isoform switch to higher *NEAT1_2* production thereby promoting the assembly of more PS.

**Figure 6.**
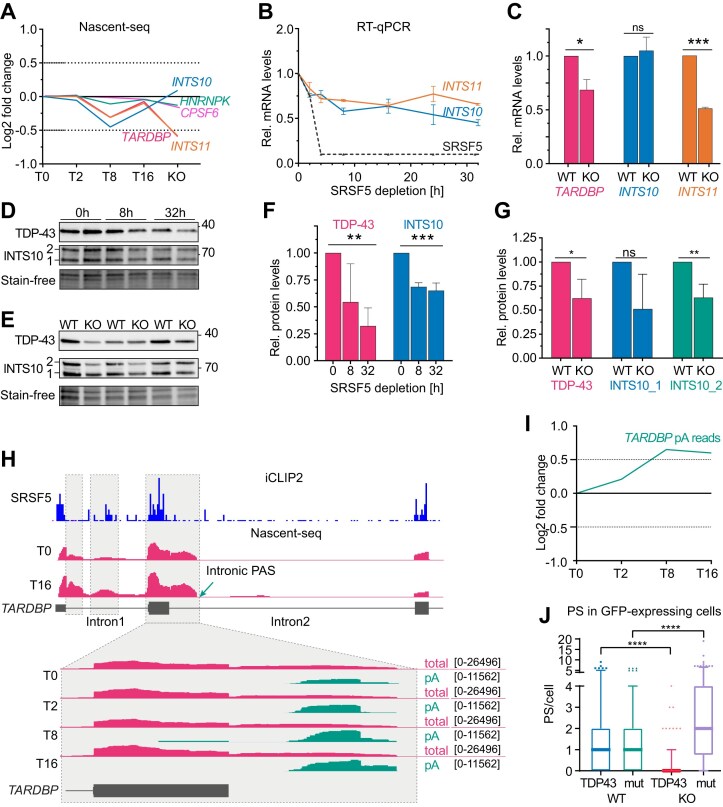
SRSF5 restricts *NEAT1_2* levels and PS assembly indirectly by regulating splicing of TDP-43. (**A**) Expression changes in genes encoding known PS regulators quantified from Nascent-seq data. (**B**) RT-qPCR of a time course of SRSF5 depletion confirms the regulation of *INTS10* and *INTS11*. (**C**) RT-qPCR of WT and SRSF5 KO cells confirms the regulation of *TARDBP*, *INTS10*, and *INTS11*. RNA levels were normalized to U6 snRNA. Graphs show mean and SD of *n* = 3 independent experiments. (**D**) Representative western blot of a time course of SRSF5 depletion showing the decrease in TDP-43 and INTS10 protein levels. Stain-free gels were used to control for equal loading. (**E**) Western blot of WT and SRSF5 KO cells (*n* = 3 replicates) showing the decrease in TDP-43 and INTS10 protein levels. Stain-free gels were used to control for equal loading. (**F** and **G**) Quantification of TDP-43 and INTS10 protein levels of the western blots partially shown in (D) and (E) from *n* = 3 experiments. (**H**) Genome browser view showing total (pink) and polyA (green) Nascent-seq reads mapping to the 5′ end of the *TARDBP* transcript from the different time points of SRSF5 depletion. SRSF5 iCLIP2 crosslinks are shown in blue. (**I**) Changes in the number of pA reads mapping to intron 2 of *TARDBP* quantified from Nascent-seq data. (**J**) Overexpression of TDP-43 but not the cytoplasmic TDP-43-mut [63, 64] decrease the number of PS in SRSF5 KO cells but not WT cells. Quantification from *n* = 3 independent replicates. Statistics: two-tailed Mann–Whitney test; *P < 0.05, **P < 0.01, ***P < 0.001, ****P < 0.0001.

The switch to *NEAT1_2* production requires enhanced PAS read-through. Several RBPs were shown to directly regulate PAS usage by binding near the *NEAT1_1* PAS and activating or inhibiting it. These include CPSF6, TDP-43, hnRNPK, and the Integrator subunits INTS10 or INTS11 [11, 21, 22]. SRSF5 probably does not act directly on the *NEAT1_1* PAS, as this region is relatively depleted of SRSF5-binding sites (Supplementary Fig. S4B). However, SRSF5 could regulate PAS usage indirectly by modulating the expression of other cleavage and polyadenylation factors, similar to SRSF3, which regulates the levels of CPSF6 by alternative splicing [60, 61]. To test this possibility, we first analyzed expression changes of known *NEAT1*/PS regulators in our Nascent-seq dataset (Supplementary Fig. S4C). We observed that transcripts encoding TDP-43 (*TARDBP)* and *INTS11* progressively decreased during SRSF5 depletion and in KO cells, whereas *HNRNPK* and *CPSF6* levels remained unchanged (Fig. 6A and Supplementary S4C). *INTS10* levels also decreased at early time points but recovered in SRSF5 KO cells (Fig. 6A). We validated the decrease of *TARDBP*, *INTS10*, and *INTS11* mRNA levels by RT-qPCR using total RNA (Fig. 6B and C, and Supplementary S4D). Moreover, protein levels of TDP-43 and INTS10 also decreased progressively in the absence of SRSF5 (Fig. 6D–F). Since both TDP-43 and INTS10 promote cleavage and termination at the *NEAT1_1* PAS [21, 22], their downregulation could enhance readthrough and *NEAT1_2* production over time.

To explore the mechanism underlying the decreased expression of *TARDBP*, *INTS10*, and *INTS11* mRNAs upon SRSF5 loss, we performed alternative splicing analysis on the Nascent-seq dataset using MAJIQ [62]. This analysis identified 23, 159, 116, and 108 significant splicing changes at T2, T8, T16, and in SRSF5 KO cells, respectively, with predominantly retained introns (Supplementary Fig. S4E). While *NEAT1*, *INTS10*, and *INTS11* showed no splicing alterations, we observed progressive retention of introns 1 and 2 in *TARDBP* transcripts following SRSF5 depletion. iCLIP2 data confirmed that SRSF5 binds to exons flanking these introns (Fig. 6H). Notably, retention of intron 2 activated an intronic PAS, resulting in premature transcript termination. Quantification of pA-containing reads mapping to the *TARDBP* locus revealed that this intronic PAS was increasingly used over time (Fig. 6H and I). These data indicate that SRSF5 normally promotes splicing of *TARDBP* introns 1 and 2. In its absence, both introns are more retained, triggering intronic PAS usage and early transcription termination at this site. Reduced production of full-length *TARDBP* mRNA decreases TDP-43 protein levels.

To directly test whether TDP-43 downregulation contributes to PS over-compensation in SRSF5 KO cells, we transiently overexpressed either WT TDP-43-GFP or a mutant version with a defective nuclear localization signal (TDP-43-NLSmut-GFP) that is less efficiently imported into the nucleus [63, 64], into WT and KO cells (Supplementary Fig. S5A). Quantification of PS number in cells expressing GFP revealed that TDP-43 overexpression strongly suppressed PS formation in SRSF5 KO cells, but had no effect in WT cells. The mislocalized TDP-43 mutant, however, did not reduce PS number or size in KO cells (Fig. 6J). PS reduction was mirrored by lower *NEAT1_2* levels measured by RT-qPCR (Supplementary Fig. S5B).

To independently demonstrate that a modest reduction in TDP-43 levels is sufficient to increase *NEAT1_2* expression and PS numbers, we generated a HeLa cell line where TDP-43 was endogenously tagged with GFP. We integrated our hGRAD system and induced TDP-43-GFP degradation by DOX (48 h and Supplementary Fig. S5C). A reduction in total TDP-43 levels by ∼50% was sufficient to increase *NEAT1_2* levels by 1.9-fold and PS numbers by 1.5-fold (Supplementary Fig. S5D–F). Together these data support a model in which long-term SRSF5 depletion results in reduced TDP-43 levels, which in turn promotes PAS read-through and enhanced production of *NEAT1_2*. A stabilization of *NEAT1_2* RNAs at later time points of SRSF5 depletion, hinted by our data (Fig. 5H), may contribute to this recovery. Higher *NEAT1_2* levels compensate for the initial loss of PS assembly over time and might constitute a feedback mechanism. Thus, SRSF5 appears to be a novel regulator of TDP-43, that sustains high TDP-43 levels and this way regulates PS assembly also in an indirect manner, by suppressing *NEAT1_2* expression.

### SRSF5 is required for PS cluster assembly during stress

**Figure 7.**
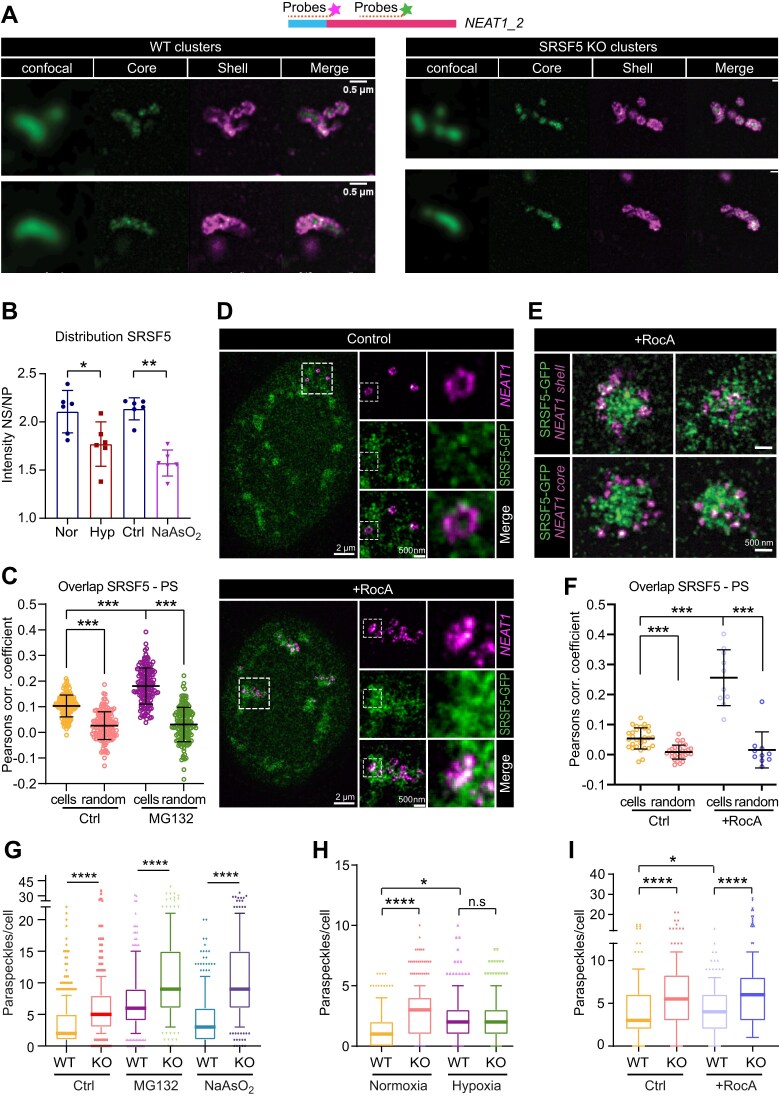
SRSF5 is required for PS cluster formation during stress. (**A**) Representative micrographs of WT and SRSF5 KO cells showing different morphologies of PS clusters—elongated rod-like structures or randomly aggregated PS spheres—using confocal and STED imaging. PS were labeled simultaneously with two different probes hybridizing to the middle region (core) or the 5′ end (shell) of *NEAT1_2*; scale bars: 500 nm. (**B**) SRSF5 is released from NS into the NP in hypoxia and after sodium arsenite treatment. Shown is the ratio of SRSF5 signal intensity in NS and a similar area of NP. (**C**) Quantification of the co-localization of PS and SRSF5 speckles in untreated (DMSO, *n* = 809 PS) and treated (MG132, *n* = 1393 PS) cells. Plotted are the Pearson correlation scores between datasets. (**D**) Representative micrographs of SRSF5-GFP cells treated with RocA (5 μM, 4 h) using STED imaging. PS were labeled with probes hybridizing to the 5′ end (shell) of *NEAT1_2*; scale bars: 2 μm and 500 nm. (**E**) STED imaging using RNA-FISH probes targeting the 5′ end (shell, top) or middle region (core, bottom) of *NEAT1_2* and SRSF5-GFP using GFP nanobodies. The panels show two examples for each staining illustrating how PS and SRSF5 speckles merge; scale bars: 2 μm and 500 nm. (**F**) Quantification of the overlap of PS and SRSF5 speckles in untreated (-RocA) and treated (+RocA) cells. Plotted are the correlation scores between datasets. (**G**) WT and SRSF5 KO cells were subjected to MG132 (10 μM, 1 h), and sodium arsenite (0.25 mM, 1 h). Quantification of PS per cell from *n* = 191 cells for MG132 and *n* = 428 cells for sodium arsenite. (**H**) WT and SRSF5 KO cells were subjected to hypoxia (0.2% O_2_, 24 h) and *n* = 300–333 cells were quantified. (**I**) WT and SRSF5 KO cells were subjected to RocA (5 μM, 4 h) and *n* = 308–387 cells were quantified. Statistics: Two-tailed Mann–Whitney Test; *P < 0.05, **P < 0.01, ***P < 0.001, ****P < 0.0001; ns = not significant.

Our data so far suggest that a small fraction of PS (18.4%) overlaps with SRSF5 speckles, and that SRSF5 promotes the assembly or stability of large PS clusters while simultaneously restricting their numbers via a TDP-43-dependent feedback loop. To investigate whether the morphology of overcompensated PS clusters differs in the absence of SRSF5, we performed STED imaging using two different RNA-FISH probe sets targeting either the middle region (core) or to the 5′ end (shell) of *NEAT1_2* (Fig. 7A and Supplementary S6A). Interestingly, we observed two distinct PS cluster architectures: the canonical elongated, cylinder-like structures and more irregular aggregates of individual PS spheres. Comparing the relative proportions of both cluster types between WT and SRSF5 KO cells (Supplementary Fig. S6B) revealed, however, no significant differences, suggesting that SRSF5 loss does not change the overall architecture of PS clusters when they are recovered.

PS clusters typically emerge when *NEAT1* levels rise dramatically, e.g. in stress conditions, and they are proposed to sequester PSPs and modulate their activities in transcription or splicing during the stress response [1, 15, 33]. To determine whether SRSF5 association with PS cluster increases under stress conditions, we examined four stressors known to induce PS clustering [33]: oxidative stress (sodium arsenite, 0.25 mM, 1 h), hypoxia (24 h 0.2% O_2_), proteasome inhibition (MG132, 10 μM, 4 h), and translation inhibition (rocaglamide A, RocA, 5 μM, 4 h). PS-SRSF5 co-localization was monitored by RNA-FISH in cells expressing SRSF5-GFP (Supplementary Fig. S6C).

Interestingly, sodium arsenite released SRSF5 almost completely from NS into the NP (Fig. 7B and Supplementary S6C), and PS are now surrounded by nucleoplasmic SRSF5 signal, while the morphology of NS was unchanged. Hypoxia also released SRSF5 from NS into the NP (Fig. 7B and Supplementary S6C), now surrounding PS spheres, while NS persist.

MG132 treatment showed a very different phenotype. It triggered the formation of large PS clusters in line with increased *NEAT1* levels (Supplementary Fig. S6C and D), but SRSF5 localization to NS was not altered. Instead, enlarged PS clusters now co-localized with SRSF5 inside of NS. Calculated PCC values were significantly higher for MG132-treated cells (∼0.18) than for untreated cells (0.1), while both values were significantly higher than those of the randomized controls (both ∼0.03; Fig. 7C). Counting of overlapping PS clusters and SRSF5 speckles revealed a significant increase from 14.0% (113/809) in untreated cells to 31.4% (437/1393) in MG132-treated cells. Both overlaps were higher than after randomization (7.5%, 61/809 and 14.8%, 207/1393, respectively) supporting the results of the PCC analysis.

The most striking change occurred upon RocA treatment. Here PS clusters completely merged with SRSF5 speckles (Supplementary Fig. S6C), which was confirmed by STED super-resolution imaging (Fig. 7D). RNA-FISH targeting either *NEAT1_2* core or shell regions revealed that PS spheres are initially still intact and decorate the periphery of SRSF5 speckles, but ultimately become fully immersed in the SRSF5 signal (Fig. 7E). PCC values calculated for SRSF5-PS image pairs increased from ∼0.01 in untreated cells to ∼0.26 in RocA cells, while both values were significantly higher than those of the randomized controls (∼0.01 e.g. ∼0.02; Fig. 7F). Supporting the PCC analysis, PS overlapping with SRSF5 clusters increased significantly from 18.4% (7/38 PS cluster) in untreated cells to 50.7% (37/73 PS cluster) in RocA treated cells. Both overlaps exceeded randomized control overlap levels (0/38 and 5/73, respectively; Fig. 7F). These results demonstrate that the nuclear mobility of SRSF5 and PS varies with the stress type, but in all cases the co-localization of SRSF5 and PS increases—either within NS or outside in the NP.

To test whether SRSF5 is necessary for PS cluster formation during stress, we exposed SRSF5 KO cells to the same four stress conditions and monitored the numbers of PS by RNA-FISH. While SRSF5 KO cells hyper-reacted to MG132 and sodium arsenite treatment, with a huge increase in PS clusters (Fig. 7G and Supplementary Fig. S7A), PS assembly was markedly impaired in hypoxia stress and after RocA treatment, compared to WT cells Fig. 7H and I. Moreover, SRSF5 KO cells formed significantly more SGs than WT cells upon sodium arsenite and MG132 treatment (Supplementary Fig. S7A and B), and differential gene expression analysis from our Nascent-seq dataset revealed a widespread misregulation of genes involved in intracellular stress signaling (Supplementary Fig. S8). Altogether, these findings suggest that although compensatory PS clusters can form in the absence of SRSF5, the assembly of new PS clusters still requires SRSF5. The lack of SRSF5 dysregulates the general stress response and renders cells more susceptible to stressors.

### SRSF5 is removed from mature PS by helicases to prevent fusion with NS

**Figure 8.**
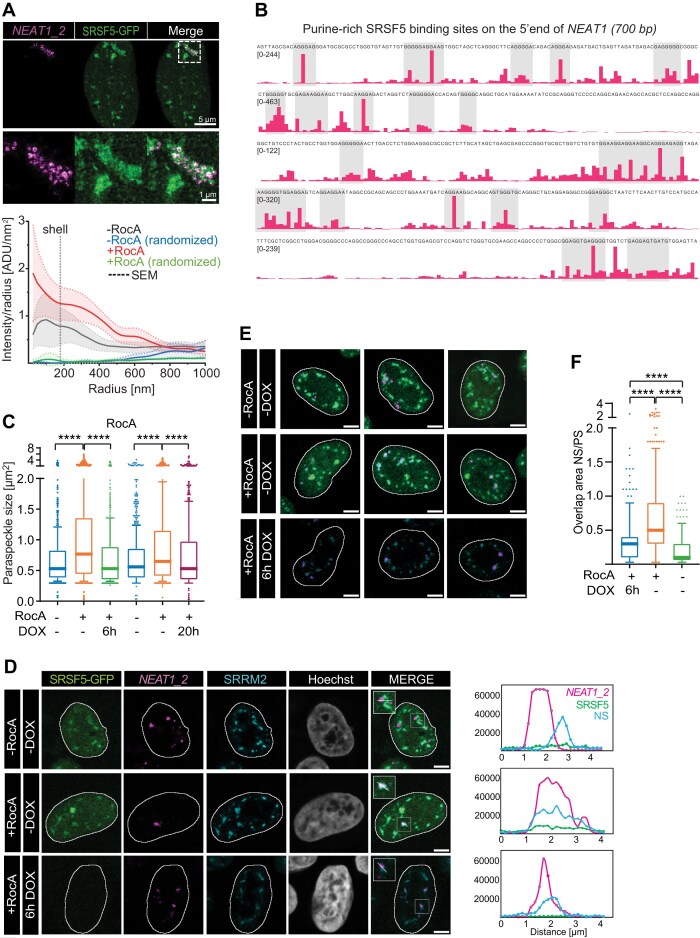
SRSF5 binding contributes to the merge of PS and NS upon RocA treatment. (**A**) Top: Super-resolution STED images of Hela SRSF5-GFP cells treated with or without RocA (4 h, 5 μM) using RNA-FISH probes targeting the 5′ end of *NEAT1_2* (shell) and GFP nanobodies targeting SRSF5-GFP; scale bars: 5 and 1 μm. Bottom: Radial plot showing averaged SRSF5 signal intensities measured within PS spheres starting from the center outwards with increasing radius. Comparison of RocA treated (4 h, 5 μM) and untreated cells (-RocA) to randomized controls. (**B**) Browser shot showing purine-rich binding sites (gray shades) of SRSF5 within the first 700 bp of the *NEAT1* transcript. (**C–** **F**) SRSF5-GFP cells were treated with RocA (4 h) and DOX (6 and 20 h, 1 μg/ml) to deplete SRSF5 simultaneously. (C) Quantification of PS cluster size from *n* = 439–724 PS. (D) Left: Example micrographs of SRSF5-GFP cells treated with RocA and DOX (6 h). PS were labeled by RNA-FISH targeting the middle region of *NEAT1_2*. NS are labeled by IF using an anti-SRRM2 antibody. Nuclei were stained with Hoechst; scale bars: 5 μm. Right: Line scans of the merged channels. (**E**) Additional examples of SRSF5-GFP cells treated simultaneously with RocA and DOX (6 h); scale bars: 5 μm. (F) Quantification of the overlap size between PS and SRSF5 speckles from control cells (*n* = 301), +Roc (*n* = 572), and +Roc & 6 h DOX (*n* = 333). Statistics: Two-tailed Mann–Whitney test, ****P < 0.0001.

Our findings suggest that a small subset of SRSF5 overlaps with the shell of PS spheres under normal conditions (Figs. 1, 3G, and 7D). To investigate this localization in more detail, we used STED imaging and quantified SRSF5 signal distribution inside and around PS spheres. To avoid diffuse background signal from nucleoplasmic SRSF5, we restricted the analysis to high-intensity SRSF5 speckles (Supplementary Fig. S1B). Radial intensity profiles of averaged SRSF5 signal intensities revealed that SRSF5 is depleted from the center of PS spheres in untreated cells but steadily accumulates in the shell region (radius: 100–180 nm) before declining towards the periphery (Fig. 8A). This distribution confirms the SRSF5 overlap with the PS shell and its immediate surroundings in a subset of PS. In RocA treated cells, this distribution is altered. SRSF5 speckle signal is generally much higher and now peaks in the PS center with a sharp decrease towards the periphery. This indicates a nearly complete spatial overlap between PS and SRSF5 speckles (Fig. 8A).

We wondered how a short RocA treatment can override the spatial segregation of PS and NS—two distinct MLOs that normally never mix [29]. RocA is known to inhibit translation, and it does so by binding to purine-rich RNA sequences and clamping the cytoplasmic RNA helicases eIF4A and DDX3X thereby preventing their action and release [65]. However, neither CHX nor amino acid starvation induced a merge between PS and NS (Supplementary Fig. S9A), indicating that the fusion of PS and SRSF5 speckles was not a consequence of translation inhibition per se.

We thus hypothesized that RocA may act in the nucleus, impairing the nuclear functions of eIF4A and DDX3X or inhibiting other nuclear helicases that remodel RBPs bound to purine-rich RNAs. Supporting this idea, our iCLIP data show that SRSF5 binds preferentially to purine-rich stretches at the 5′ end of *NEAT1* (Fig. 8B). To directly test whether SRSF5 contributes to the RocA-induced merge of PS and NS, we used SRSF5 hGRAD cells and depleted SRSF5 (6 and 20 h) simultaneous to RocA treatment (4 h). Acute depletion of SRSF5 impaired RocA-induced PS cluster formation (Fig. 8C and Supplementary Fig. S9B), but more importantly, PS that were fused with NS after RocA treatment slightly moved apart when SRSF5 was acutely depleted (6 h), compared to control cells (Fig. 8D and Supplementary Fig. S9C). Indeed, quantification of the NS–PS overlap sizes showed a significant decrease when SRSF5 is removed (Fig. 8E and F).

These results support a model where SRSF5 binds transiently to nascent *NEAT1* transcripts during early stages of PS formation, but is later removed from mature PS by helicases, likely as part of normal PS remodeling. RocA, by clamping helicases to the purine-rich 5′ end of *NEAT1* RNA, traps SRSF5 on mature PS and prevents its removal. Failure to remove SRSF5 from PS shells eventually targets PS to NS, where SRSF5 normally resides, ultimately leading to the immersion of both MLOs. Our data suggest that PS are continuously remodeled by RNA helicases, particularly in response to stress. SRSF5 perhaps together with other NS-resident proteins, appears to function as a transient architectural factor during early PS assembly, but its timely removal seems to be essential to maintaining the spatial identity and segregation of nuclear MLOs.

## Discussion

We show here that PS and NS engage in dynamic crosstalk and exchange components during cellular homeostasis and stress conditions. Specifically, we found that the NS-resident SRSF5 is required for efficient PS assembly and cluster formation in response to stress. Our data suggest that a subset of PS overlap with SRSF5 in their shell regions, and that SRSF5 preferentially binds to purine-rich stretches at the 5′ end of *NEAT1* RNA. This binding appears to promote *NEAT1* transcription, proper alignment of *NEAT1_2* 5′ ends within the PS shell, and the formation of larger PS clusters, all of which is important for the cellular stress response. In contrast, acute depletion of SRSF5 leads to decreased *NEAT1_2* levels, misalignment of *NEAT1* 5′ ends, smaller PS spheres, and impaired cluster formation.

Consistent with estimates that ∼10%–30% of PS represent nascent structures that are still closely associated with the *NEAT1* transcription site [7, 12, 18], we observed that ∼18% of PS overlap with SRSF5 speckles. This partial colocalization suggests that SRSF5 binding to *NEAT1* might be transient and affect only a small fraction of nascent PS during early assembly. The mechanistic role of SRSF5 here could be multifaceted. (i) Our nascent RNA-seq and RNA stability data point to a transcriptional role for SRSF5. One possibility could be that SRSF5 stimulates the release of RNA polymerase II (Pol II) from promoter-proximal pausing, a mechanism that was demonstrated for SRSF2 [66]. SRSF2 frequently binds together with SRSF5 on transcripts and shares overlapping functions [50]. Indeed, our iCLIP data show that SRSF5 binds also very strongly to 7SK RNA (data not shown), which is part of the inhibitory complex sequestering the transcription activator kinase P-TEFb [66]. Moreover, the 7SK methylphosphate capping enzyme (MECPE), which is part of the 7SK RNP, is strongly enriched in our SRSF5 proximity MS dataset. By transitioning from the 7SK RNP to purine-rich stretches at the 5′ end of *NEAT1* RNA during transcription, SRSF5 may release Pol II from pausing. We also observed a strong spatial enrichment of ZNF326 in our proximity-MS dataset, which is a component of the DBIRD complex that integrates transcription elongation with alternative splicing of large exons [67]. (ii) Alternatively, or additionally, SRSF5 binding in the PS shell may provide a protective environment for *NEAT1* 5′ ends, which are more exposed to the NP. This protection could prevent unwanted splicing, modification, or decay, thus ensuring the proper maturation of PS. (iii) Finally, SRSF5 may also play a role in PS architecture by organizing the different layers of PS and helping align *NEAT1* 5′ ends within the PS shell. A similar function has been proposed for other PS shell proteins, and it was shown that their competitive binding with PS core proteins to overlapping regions within *NEAT1* transcripts and their immiscibility organizes PS layers during assembly [68]. This idea is supported by our findings that SRSF5 preferentially binds to the 5′ end of *NEAT1* transcripts and overlaps with the shell of a subset of PS. In its acute absence, *NEAT1* 5′ ends move inwards, towards the core of PS. Moreover, NS components including SRSF5 usually do not mix with PSPs [27].

Acute depletion of SRSF5 led to a transient disappearance of PS followed by a later overcompensation. In contrast, acute depletion of NONO also disassembled PS but many small *NEAT1_2*-positive foci persisted, consistent with previous observations [7]. This phenotypic difference also suggests that in addition to an architectural role in PS assembly, SRSF5 may also promote *NEAT1* transcription. While depletion of SRSF5 or NONO disrupts PS assembly and cluster formation, *NEAT1* transcripts continue to be produced in NONO-depleted cells.

Our data further suggest that SRSF5 must be actively removed from the shell after maturation to prevent unwanted fusion with NS once mature PS are released from chromatin. This removal is likely mediated by nuclear RNA helicases. Supporting this model, we find many nuclear helicases that are enriched in our SRSF5 proximity MS dataset including eIF4A3. Moreover, we show that RocA, a compound which clamps helicases such as eIF4A1/2 and DDX3X onto purine-rich RNA [65], induces the fusion of PS and NS, while acute depletion of SRSF5 partially reverses this phenotype. These findings support the idea that normal PS remodeling is prevented by RocA, and that aberrant retention of SRSF5—and may be other NS-residents—in the shell of PS contributes to the merging of the two MLOs. The partial fusion of PS and NS observed after proteasome inhibition is also interesting and suggest that remodeling involves ubiquitination and degradation of the removed RBPs. A dynamic PS remodeling would also explain why only 18% of PS overlap with SRSF5 speckles at steady state, and why SRSF5 KO cells are able to form overcompensated PS cluster lacking SRSF5 in their shells. Still, SRSF5 KO cells remain highly susceptible to stress, as *de novo NEAT1* transcription and formation of new PS clusters still require SRSF5.

A recent study demonstrated that the 5′ end of *NEAT1* and the specific composition of RBPs binding to the shell region are crucial for maintaining the segregation between PS and NS [29]. It was proposed that RBPs residing in the shell of PS and those within NS differ fundamentally in their chemical and biophysical properties, for instance, proteins with prion-like domains such as SFPQ versus proteins with mixed charge domains such as SR proteins—thereby increasing the energy barrier at the PS-NS interface [29]. Consistent with this, depletion of PS shell proteins including SFPQ, HNRNPF, and BRG1 promoted the fusion of PS and NS [29]. Here, we show that acute depletion of SRSF5 caused the opposite effect, increasing the distance between PS and NS. Conversely, RocA treatment caused a completely merge of SRSF5, NS, and PS, a phenotype that was partially reversed by acute SRSF5 depletion. This aberrant fusion seems to be analogous to the artificial tethering of U2 snRNP proteins that normally reside in NS, to the PS shell region of *NEAT1*, which also drove the internalization of PS within NS [29]. Based on this, we propose that permanent clamping of SRSF5, and likely other NS-residents, onto purine-rich RNA stretches at the 5′ end of *NEAT1* decorates PS shells with mixed charge domain proteins that predisposes PS to aberrantly coalesce with NS once they are liberated from chromatin.

Persistent clamping of SRSF5 to *NEAT1* would also interfere with the recruitment of shell proteins during maturation that are normally required for the separation between PS and NS. Indeed, some of the known shell proteins were absent in the SRSF5 proximity MS dataset, including BRG1 and TAF15 [29, 69], which exhibit a *NEAT1* binding profile similar to SRSF5. We hypothesized that the shells of overcompensated PS in SRSF5 KO cells are occupied by these competing proteins, which might alter the properties of PS spheres and favor clusters. Indeed, we observed two distinct types of PS clusters, the expected elongated rod-shaped PS, but also aggregates of PS spheres that assemble larger structures. However, the ratios of both cluster types were similar in WT and SRSF5 KO cells suggesting that the shell properties of overcompensated PS are similar to normal mature PS, and both lack SRSF5 in their shells, the latter due to remodeling. Rod-shaped PS arise when PS shells grow in one dimension [23] or through the fusion of individual PS spheres [70]. In contrast, the irregular aggregates of PS spheres we observed do not seem to fuse and may result from a slightly altered shell composition. These clusters may represent a form of higher-order PS organization, potentially allowing for the sequestration of distinct RBPs and RNAs, while maintaining the energetically favorable state of separated spheres. But this remains to be investigated.

Using our newly developed acute depletion system, hGRAD, we also uncovered an intriguing compensatory mechanism for PS regulation, which revealed SRSF5 as a novel regulator of TDP-43 levels. Our data suggest that SRSF5 binding to the *TARDBP* transcript normally promotes splicing of introns 1 and 2. Acute loss of SRSF5 leads to the retention of these introns, which activates an intronic polyA site (PAS) within intron 2 and results in premature transcriptional termination. Reduced production of full-length *TARDBP* mRNA decreases TDP-43 protein levels, which in turn promotes *NEAT1* PAS read-through and drives the switch to the long isoform *NEAT1_2* resulting in increased PS formation [22, 71]. This model is supported by our findings that TDP-43 overexpression in SRSF5 KO cells but not of a mutant that localizes to the cytoplasm, reduces PS. Moreover, a modest depletion of TDP-43 using hGRAD was sufficient to double *NEAT1_2* levels and PS numbers. Our data suggest that during compensation, *NEAT1_2* levels increase relatively early, through transcriptional recovery, a switch to the long isoform, and potentially enhanced RNA stability. PS numbers and sizes seem to recover only at later time points, but this might be due to resolution limits. PS spheres are small and difficult to detect by confocal microscopy. Only later, when they coalesce into larger clusters, they become more readily detectable.

Thus, our study identifies SRSF5 as a direct and also indirect regulator of PS assembly, by sustaining high TDP-43 levels and this way suppressing *NEAT1_2* expression. The compensatory feedback loop buffers the loss of SRSF5 by TDP-43 downregulation, allowing *NEAT1_2* levels to rise and PS numbers to recover and could serve several purposes: (i) it maintains PS homeostasis under non-stress conditions when SRSF5 levels fluctuate; (ii) it allows cells to compensate for the absence of SRSF5 in the long term, albeit at the cost of a dysregulated stress response, or (iii) it helps to coordinate cell fate decisions [22]. We have previously shown that SRSF5 promotes the maintenance of pluripotency by exporting transcripts encoding pluripotency factors. This activity that is blocked when cells undergo differentiation and SRSF5 is inactivated [46]. Notably, this shuttling switch correlates with the onset of *NEAT1_2* expression. TDP-43 also promotes pluripotency by repressing the formation of the long *NEAT1_2* isoform. During differentiation, TDP-43 levels decrease, *NEAT1_2* is produced, and PS begin to form. These PS now sequester existing TDP-43 and inactivate it, further shifting the balance towards *NEAT1_2* processing and promoting exit from pluripotency. Our findings suggest that SRSF5 may contribute to this cell fate decision by limiting TDP-43 levels during differentiation. SRSF5 inactivation during differentiation would block *TARDBP* splicing and SRSF5-dependent RNA export, lowering TDP-43 protein levels, facilitating the exit from pluripotency.

Our work highlights that acute depletion systems are essential to distinguish direct from indirect or compensatory effects, as RBPs always act within large compensatory networks. Furthermore, it emphasizes the need for multi-color labelling approaches and superior resolution microscopy to accurately capture dynamic conformational changes, structural defects or the precise architecture of PS clusters. Finally, our findings also carry broader biomedical implications. Dysregulation of condensate dynamics or SRSF5 levels contribute to the development and progression of tumors through the misregulation of alternative splicing and gene expression [32, 72, 73]. Understanding the precise mechanisms for their assembly and crosstalk during stress is crucial for therapeutic intervention and cancer diagnosis. Our model suggests that nuclear condensate remodeling requires helicase activity, and that inhibition of this remodeling by RocA perturbs MLO identity and function. This previously unrecognized activity of RocA could contribute to off-target consequences and might underly its failure in clinical trials despite promising anti-leukemia properties. Conversely, this additional effect of RocA could explain why other translation inhibitors did not show the same effects against leukemia.

In summary, our study proposes SRSF5 as a coordinator of nuclear condensate crosstalk by controlling *NEAT1* transcription and PS architecture, and by indirectly modulating TDP-43 levels. Our results indicate that active remodeling by helicases is essential to maintain MLO identity, and that perturbing this process disrupts nuclear organization. These insights uncover fundamental principles of nuclear body dynamics.

## Supplementary Material

gkaf713_Supplemental_Files

## Data Availability

The hGRAD plasmid is available from Addgene (Plasmid #207837). The nascent-seq and iCLIP data are available in the Gene Expression Omnibus (GEO) with the accession numbers GSE229324 (SRSF5 acute depletion), GSE288960 (SRSF5 KO), and GSE229325 (SRSF5 iCLIP2 in HeLa cells). All other data are available from the corresponding authors upon request.
