# Supplementary material for: Rapid depletion and super-resolution microscopy reveal dual roles of SRSF5 in coordinating nuclear speckle–paraspeckle crosstalk during cellular stress
Source: Nucleic Acids Res. 2025 Jul 26;53(14):gkaf713. doi: 10.1093/nar/gkaf713 (PMC13223733; doi:10.1093/nar/gkaf713)
Supplement: gkaf713_Supplemental_Files [file gkaf713_supplemental_files.zip › Arnold_Supplementary_text_revision_NAR_updated.pdf]

Rapid depletion and super-resolution microscopy reveal dual roles of SRSF5 in coordinating nuclear speckle–paraspeckle crosstalk during cellular stress

Ellen Kazumi Okuda[1,2,§], Laurell Fridolin Kessler[3,§], Benjamin Arnold[1,§], Ricarda J. Riegger[1], Maria Clara Hernández Cañás[1,4], Ewelina Zebrowska[1], Cem Bakisoglu[1,4], Mara Rudigier[1], Christine Krost[1], Helder Y. Nagasse[1], Jan Keiten-Schmitz[5], Stefan Müller[5], David Stanek[6], Dorothee Dormann[7,8], Kathi Zarnack[1,4], Mike Heilemann[3,#] & Michaela Müller-McNicoll[1,9#]

[1]Institute of Molecular Biosciences, Goethe University, Frankfurt am Main, Germany

[2]IMPRS on Cellular Biophysics

[3]Institute of Physical and Theoretical Chemistry, Goethe University, Frankfurt am Main, Germany

[4]Buchmann Institute for Molecular Life Sciences (BMLS), Frankfurt am Main, Germany

[5] Institute of Biochemistry II, Goethe University Frankfurt, Frankfurt am Main, Germany

[6]Institute of Molecular Genetics (IMG), CAS, Prague, Czech Republic

[7]Institute for Molecular Physiology, Johannes Gutenberg University (JGU), Mainz, Germany

[8]Institute of Molecular Biology (IMB), Mainz, Germany

[9]Max Planck Institute for Biophysics, Frankfurt am Main, Germany

Supplemental material

Content:

Supplementary Figures......2

Supplementary Tables......11

Supplementary Figures

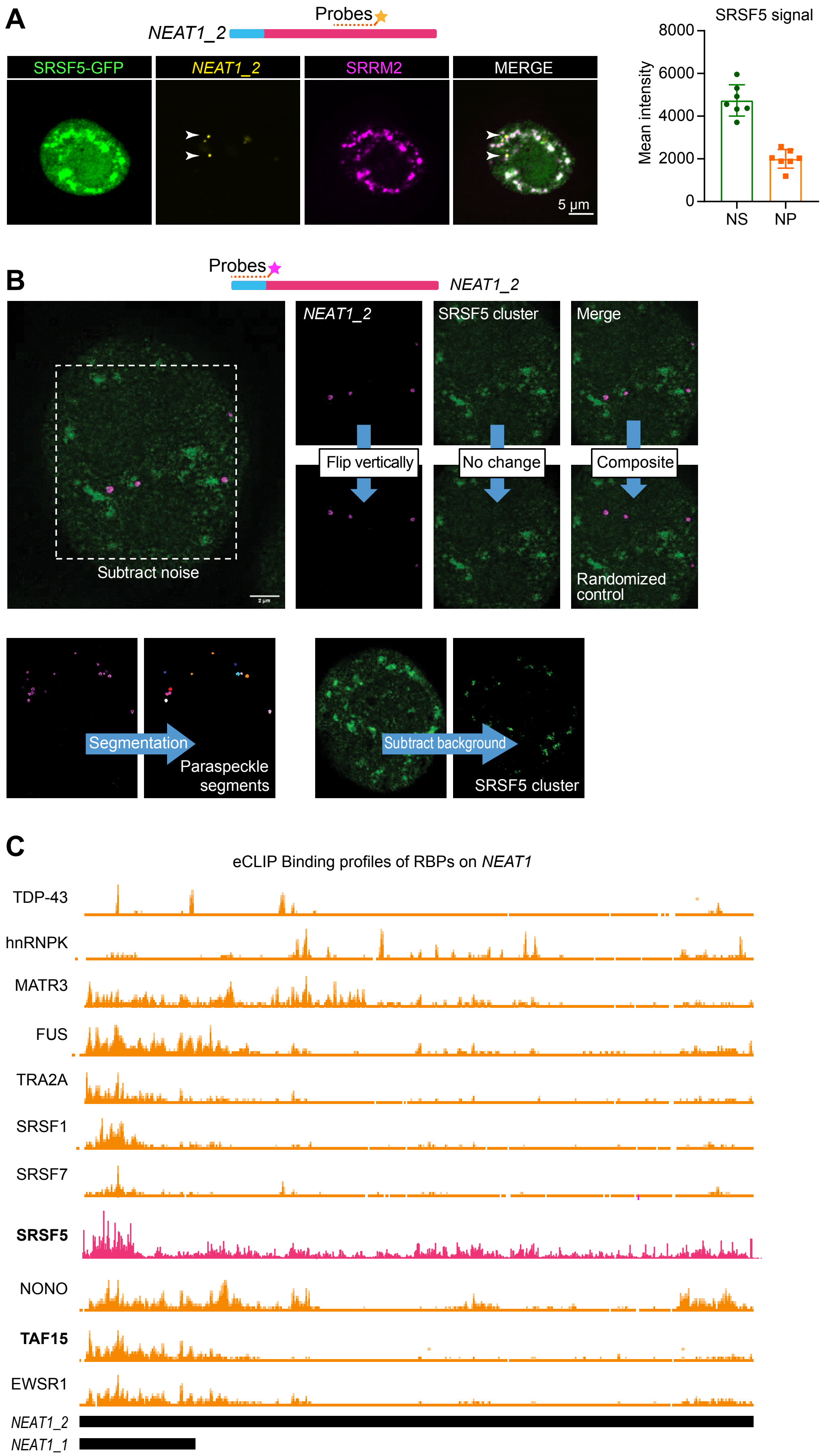

Figure S1: SRSF5 localizes to NS and PS and binds to the 5'end of *NEAT1*. A) Left: Confocal images of HeLa SRSF5-GFP cells showing that SRSF5 localizes to NS, labelled with an anti-SRRM2 antibody. PS were labelled by RNA-FISH targeting the middle region of *NEAT1_2*. **Right:** Quantification of SRSF5 signal in NS and in a similar area of the nucleoplasm (NP) reveal a ratio of 2.4 : 1. **B)** Workflow for the generation of randomized controls for the quantification of PS-SRSF5 overlap using STED microscopy. **C)** Browser shot comparing the binding profiles of SRSF5 obtained by iCLIP2 (1) and other known PSPs derived by eCLIP (2).

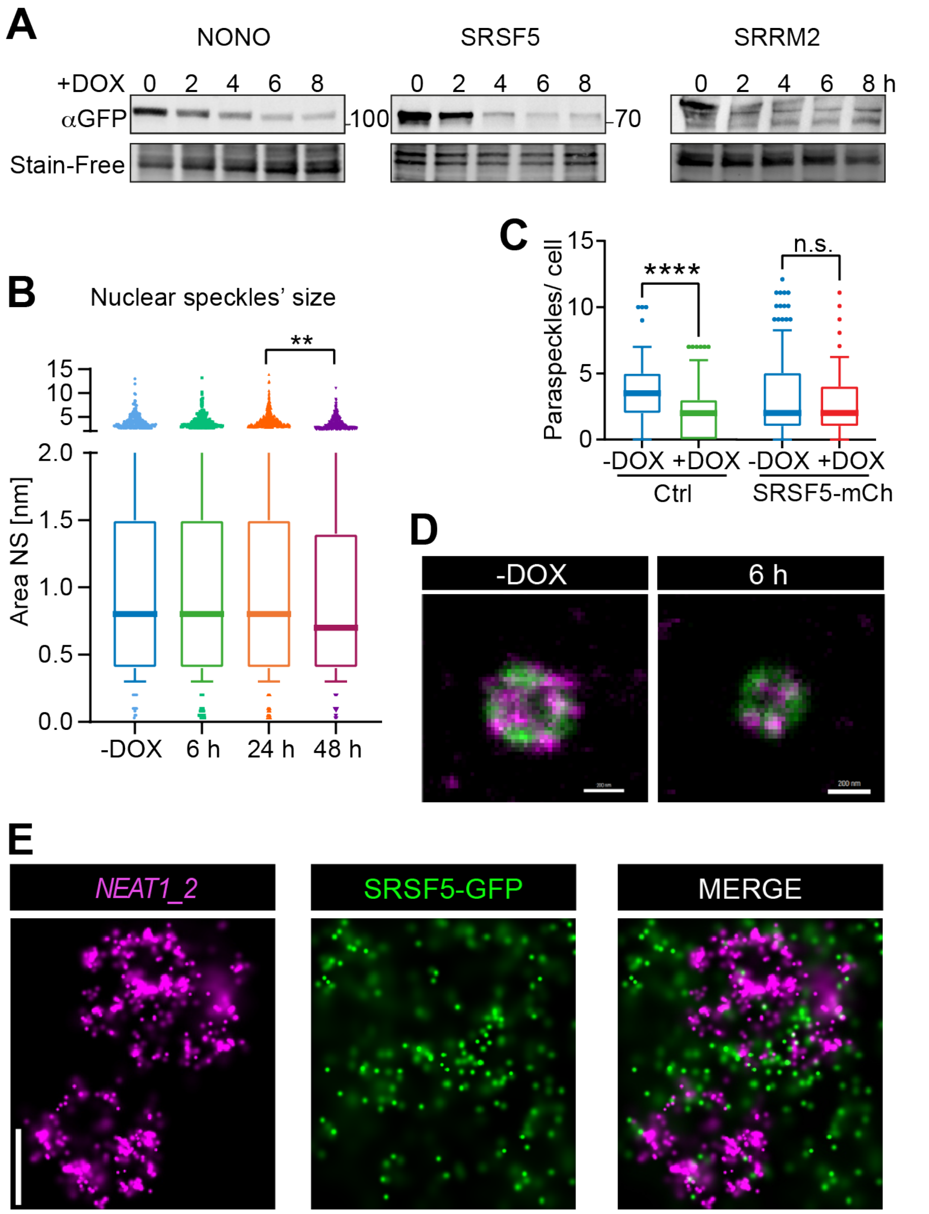

Figure S2. Paraspeckles are smaller in diameter and differently packaged upon acute depletion of SRSF5. A) Representative Western blots of the degradation timeline of NONO-, SRSF5- and SRRM2-GFP after induction of hGRAD by DOX (1 µg/mL) for 8 h. Stain-free membranes were used to control for equal loading**. B)** Quantification of nuclear speckle (NS) area in HeLa SRSF5-GFP hGRAD upon SRSF5 depletion. (-DOX, n = 1801; 6 h, n = 2739; 24 h, n = 3053; 48 h n = 2967). **C)** To rescue the phenotype, SRSF5-GFP hGRAD cells were transiently transfected with a plasmid encoding SRSF5-mCherry (24 h). SRSF5-GFP was simultaneously depleted by DOX induction (8 h) and PS were counted in control cells (-DOX=112; +DOX=152) and SRSF5-mCherry expressing cells (-DOX=295; +DOX=115). **D)** STED images of one representative PS sphere at 0 h and at 6 h SRSF5 depletion using RNA-FISH labels to target the 5' and 3'end of *NEAT1_2*. Scale bars - 200 nm. **E)** dSTORM/DNA-PAINT image of one representative PS sphere labelled at the 5'end of *NEAT1_2* using RNA-FISH probes and DNA-labelled GFP nanobodies targeting SRSF5-GFP. Scale bars - 200 nm. Statistics: Mann-Whitney test. pValue < 0.01 (**), pValue < 0.0001 (****).

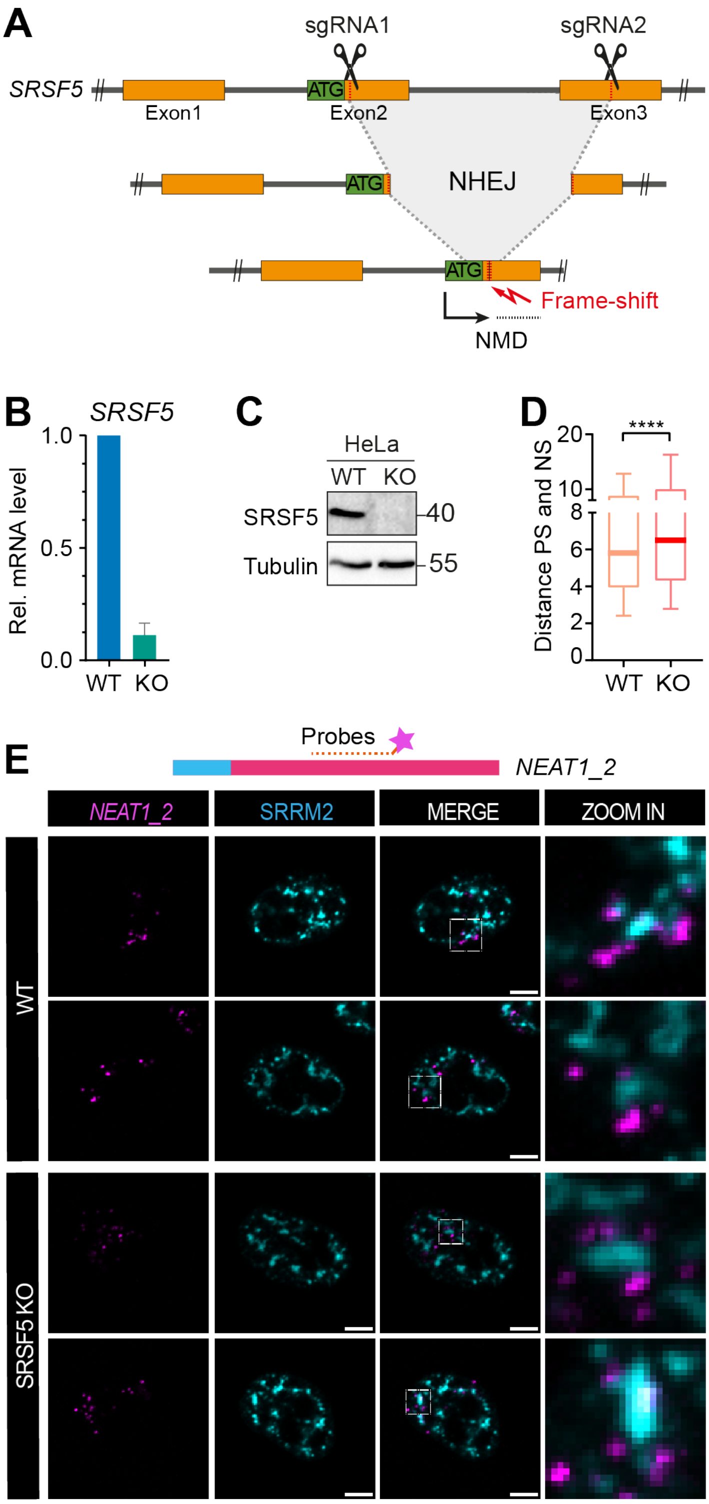

Figure S3. Long-term depletion or SRSF5 KO triggers a PS compensation mechanism. A) Generation of a HeLa SRSF5 KO cell line using CRISPR/Cas9. CRISPR genome-editing strategy using two sgRNAs to generate a frame-shift in exon 2 of the *SRSF5* gene and prevent its expression. Nonsense-mediated decay (NMD) degrades the aberrant RNA. **B)** Validation of protein KO by Western blot using an SRSF5-specific antibody (a-SRp40). Anti-tubulin was used to control for equal loading. **C)** Validation of *SRSF5* mRNA degradation by NMD using RT-qPCR quantifying the *SRSF5* levels relative to U6 snRNA. **D)** Quantification of the distance (pixel) between each PS center of mass to the closest NS center of mass for WT (n = 1275) and SRSF5 KO cells (n = 2055). **E)** Example micrographs of WT and SRSF5 KO cells. PS were labelled with probes hybridizing to the middle region of *NEAT1_2*. NS were immunostained using an anti-SRRM2 antibody. Right panels: Zoom-ins of white squares. Scale bars = 5 μm. Statistics: Mann-Whitney test. pValue < 0.0001 (****).

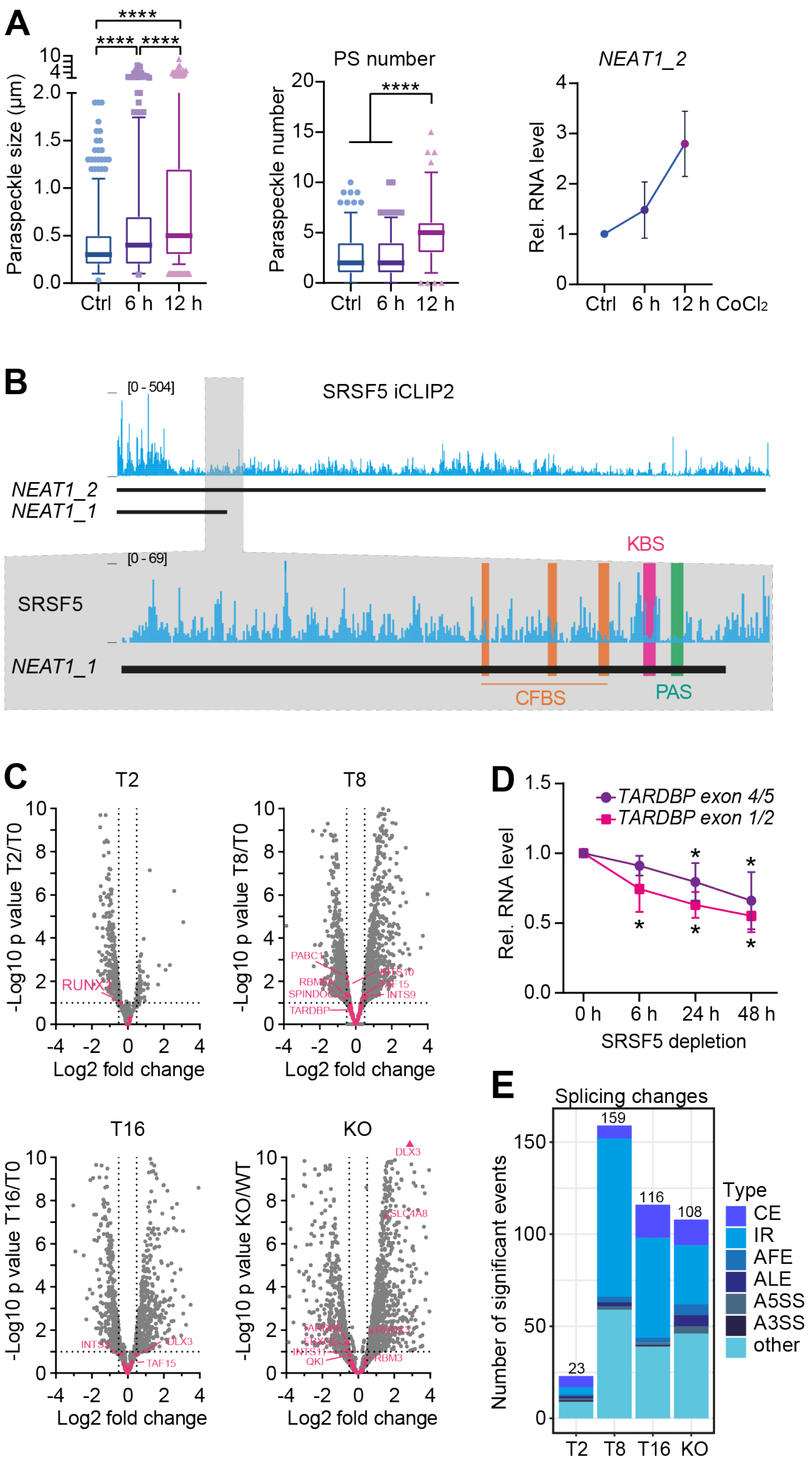

Figure S4. SRSF5 restricts *NEAT1_2* levels and PS assembly indirectly by regulating TDP-43 and INTS10. A) Quantification of *NEAT1_all* and PS cluster size after $CoCl_2$ treatment (250 µM, 6 h, 12 h) confirm a direct correlation between *NEAT1* transcriptional output and PS cluster size. **B)** Browser shots showing SRSF5 crosslinks on *NEAT1_2* RNA. Zoom-in on the poly(A) site (PAS, green) with known cleavage factor binding sites (CFBS, orange) or hnRNPK binding sites (KBS, pink). **C)** Volcano plots showing differentially expressed genes from the Nascent-seq data at T2, T8 and T16 compared to T0, or SRSF5 KO compared to WT. All genes encoding known PS regulatory factors are indicated. **D)** RT-qPCR of a time course of SRSF5 depletion confirms the down-regulation of *TARDBP* transcript using two different primer pairs. RNA levels were normalized to U6 snRNA. Graphs show mean and SD of n=3 independent experiments. **E)** Splicing changes quantified with MAJIQ from the Nascent-seq data at T2, T8 and T16 compared to T0, or SRSF5 KO compared to WT. CE - cassette exon, IR - intron retention, AFE - alternative first exon, ALE - alternative last exon, A5SS - alternative 5'splice site, A3SS - alternative 3'splice site. Statistics: two-tailed Mann-Whitney Test. * $p < 0.05$; ** $p < 0.01$.

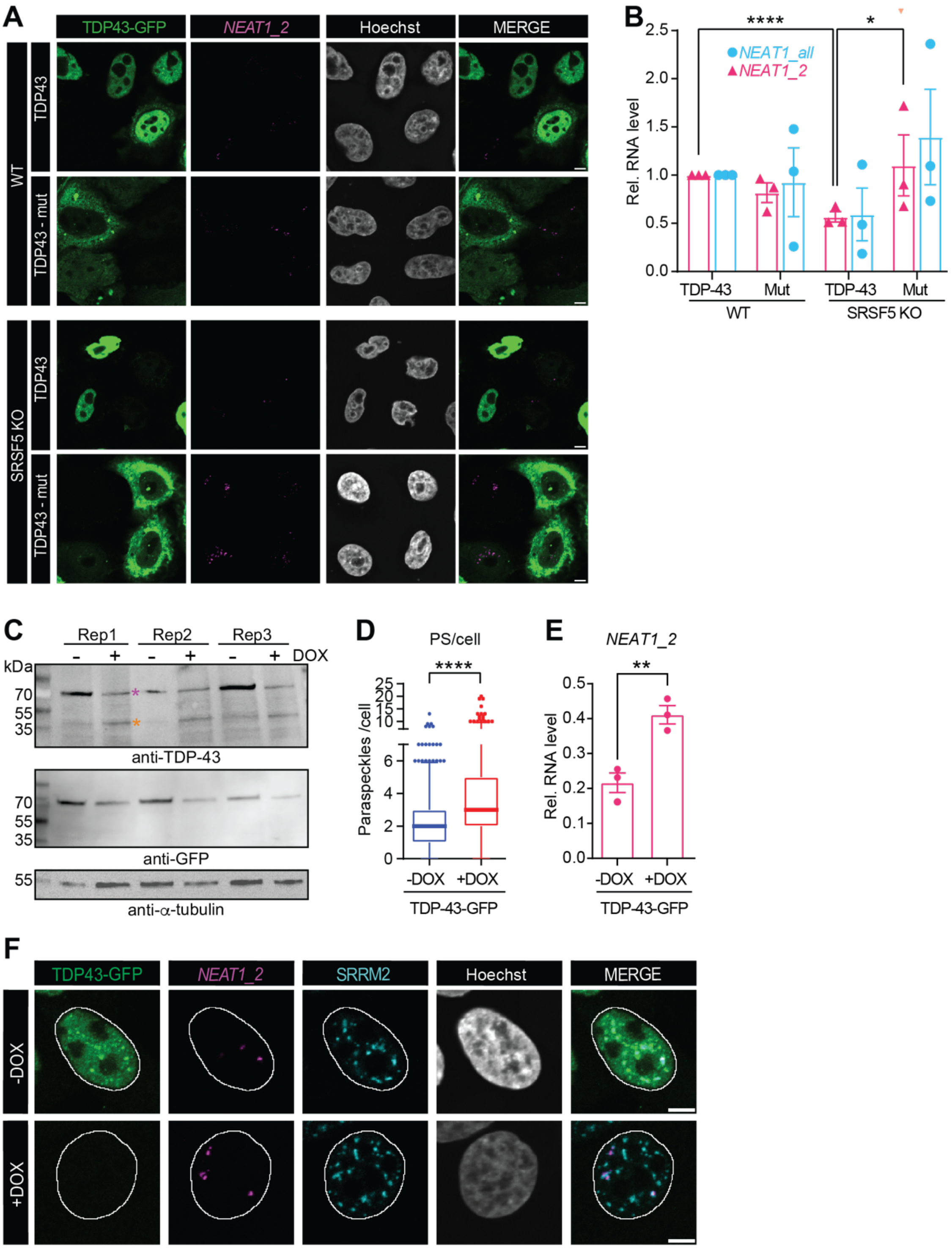

Figure S5. TDP-43 regulates *NEAT1_2* expression and PS cluster assembly. A) Representative micrographs of HeLa WT and SRSF5 KO cells after transient transfection with GFP-tagged TDP-43 (WT) and a mutant version (TDP43-NLSmut). TDP43-NLSmut is mutated in the nuclear localization signal (NLS) of TDP-43 (K82A, R83A, K84A, K95A, K96A, R97A) and accumulates in the cytoplasm. **B)** RT-qPCR to quantify *NEAT1_2* and *NEAT1_all* levels in WT and SRSF5 KO cells transiently transfected with WT TDP-43 or TDP43-NLSmut. **C)** Western blot using anti-TDP-43, anti-GFP and anti-α-Tubulin antibodies to monitor depletion of endogenously tagged TDP-43-GFP by DOX induction (48 h, 1 μM/ml, n=3 replicates). **D)** Quantification of PS number in control cells (n=367) and after depletion of TDP-43 (n=441). **E)** Quantification of *NEAT1_2* levels by RT-qPCR. **F)** Example micrographs of TDP-43-GFP cells after DOX induction (48 h). NS were immuno-stained using an anti-SRRM2 antibody. Nuclei were stained with Hoechst. PS were labelled with probes hybridizing to the middle region of *NEAT1_2*. Scale bars = 5 μm. RNA levels were normalized to U6 snRNA. Graphs show mean and SD of n=3 independent experiments. Statistics: two-tailed Mann-Whitney Test. * $p < 0.05$; ** $p < 0.01$; *** $p < 0.001$; **** $p < 0.0001$.

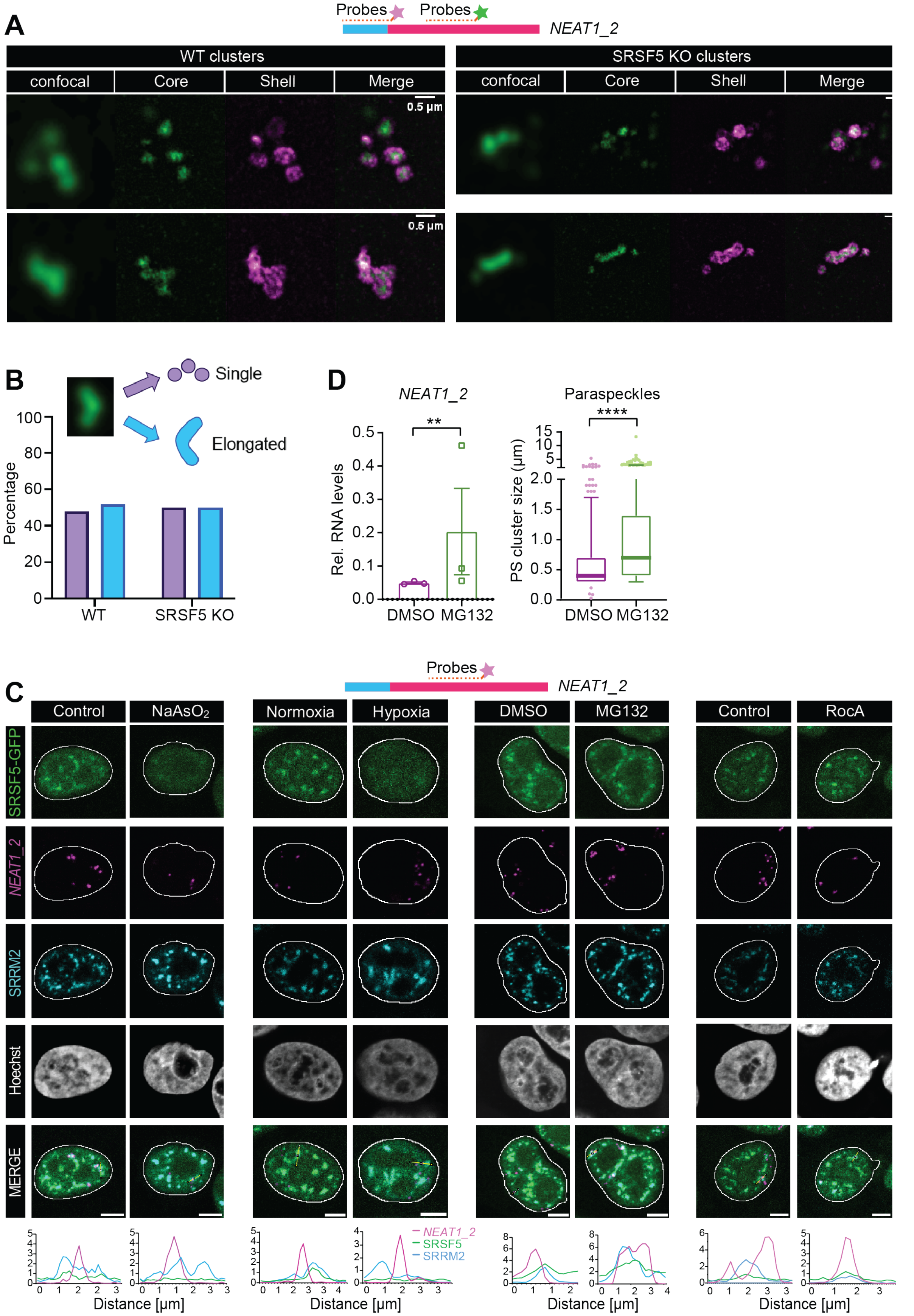

Figure S6: Proximity between SRSF5 and PS is altered dependent on the stress conditions. A) Representative micrographs of WT and SRSF5 KO cells showing the different morphologies of PS clusters - elongated rod-like structures or aggregated PS spheres - using confocal and STED imaging. PS were labelled with probes hybridizing to the middle region (core) or the 5'end (shell) of *NEAT1_2*. Scale bars = 500 nm. **B)** Determination of the ratio of

elongated rods versus aggregated PS spheres in WT and SRSF5 KO cells from n = 40 cells. **C) Top:** Confocal images of HeLa SRSF5-GFP cells subjected to sodium arsenite (1 h, 0.25 mM), hypoxia (24 h, 0.2% O_2, 5% CO_2), MG132 (4 h, 10 µM) and RocA (4 h, 5 µM). PS were labelled by RNA-FISH targeting the middle region of *NEAT1_2*. NS are labelled by IF using an anti-SRRM2 antibody. Nuclei were stained with Hoechst. Scale bars 5 µm. **Bottom:** Example line scans show co-localization between SRSF5 signal and PS. **D)** WT cells treated with DMSO or MG132 (4 h, 10 µM). **Left:** RT-qPCR to quantify *NEAT1_2* levels. RNA levels were normalized to U6 snRNA. Graphs show mean and SD of n=3 independent experiments. **Right:** Quantification of PS cluster size. Statistics: two-tailed Mann-Whitney Test. * $p < 0.05$; ** $p < 0.01$; *** $p < 0.001$; **** $p < 0.0001$.

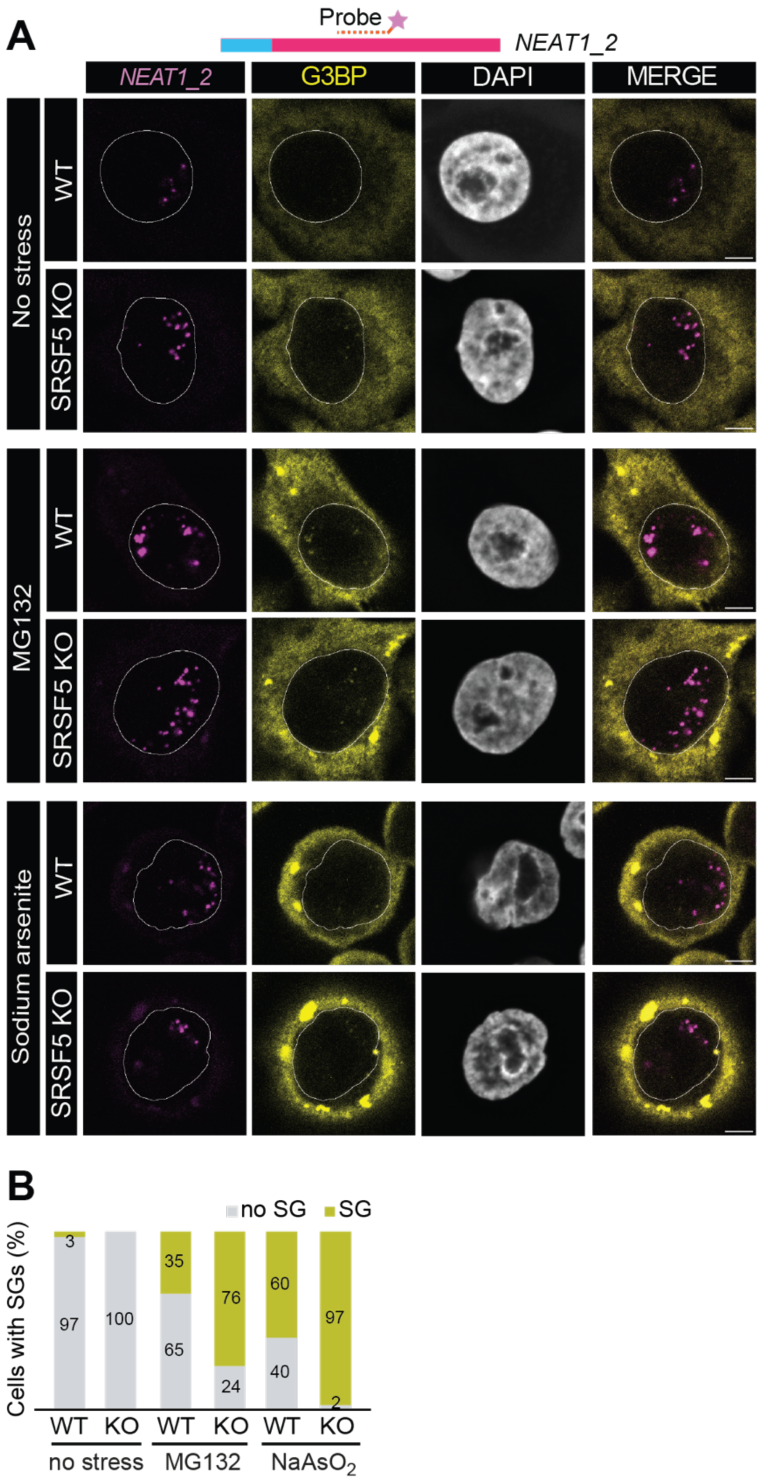

Figure S7: SRSF5 KO cells are more sensitive to stress. A) Example micrographs of HeLa WT and SRSF5 KO cells subjected to MG132 (4 h, 10 µM) and sodium arsenite (4 h, 0.25 mM). PS are labelled by RNA-FISH with a probe hybridizing to the middle region of *NEAT1_2*. SGs are labelled by immunofluorescence (IF) using an anti-G3BP1 antibody. Nuclei are stained with Hoechst. Scale bars - 5 µm. **B)** Quantification of stress granules SGs.

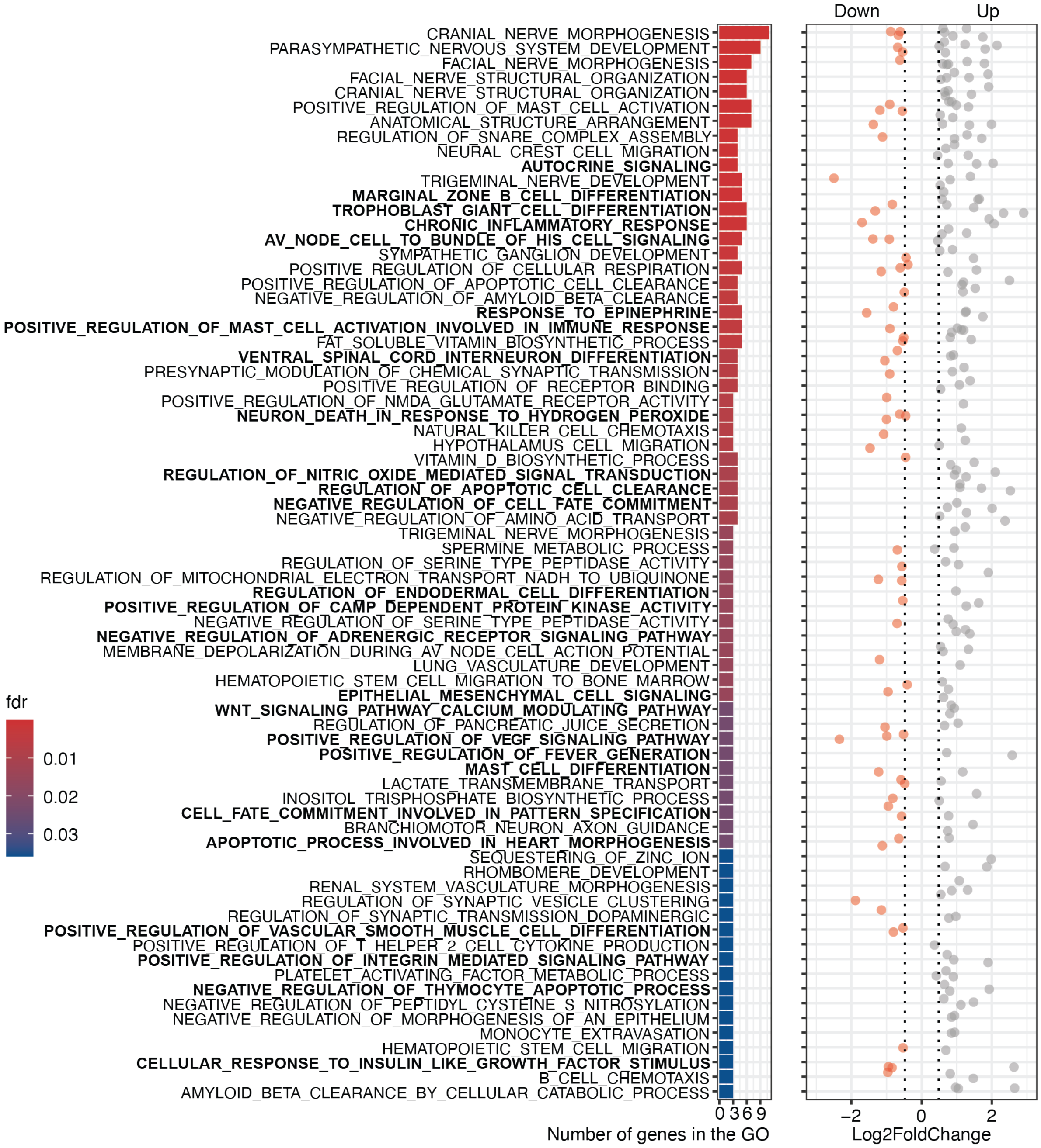

Figure S8: GO-term analysis of significantly differentially expressed genes indicates the dysregulation of many signalling pathways in the absence of SRSF5.

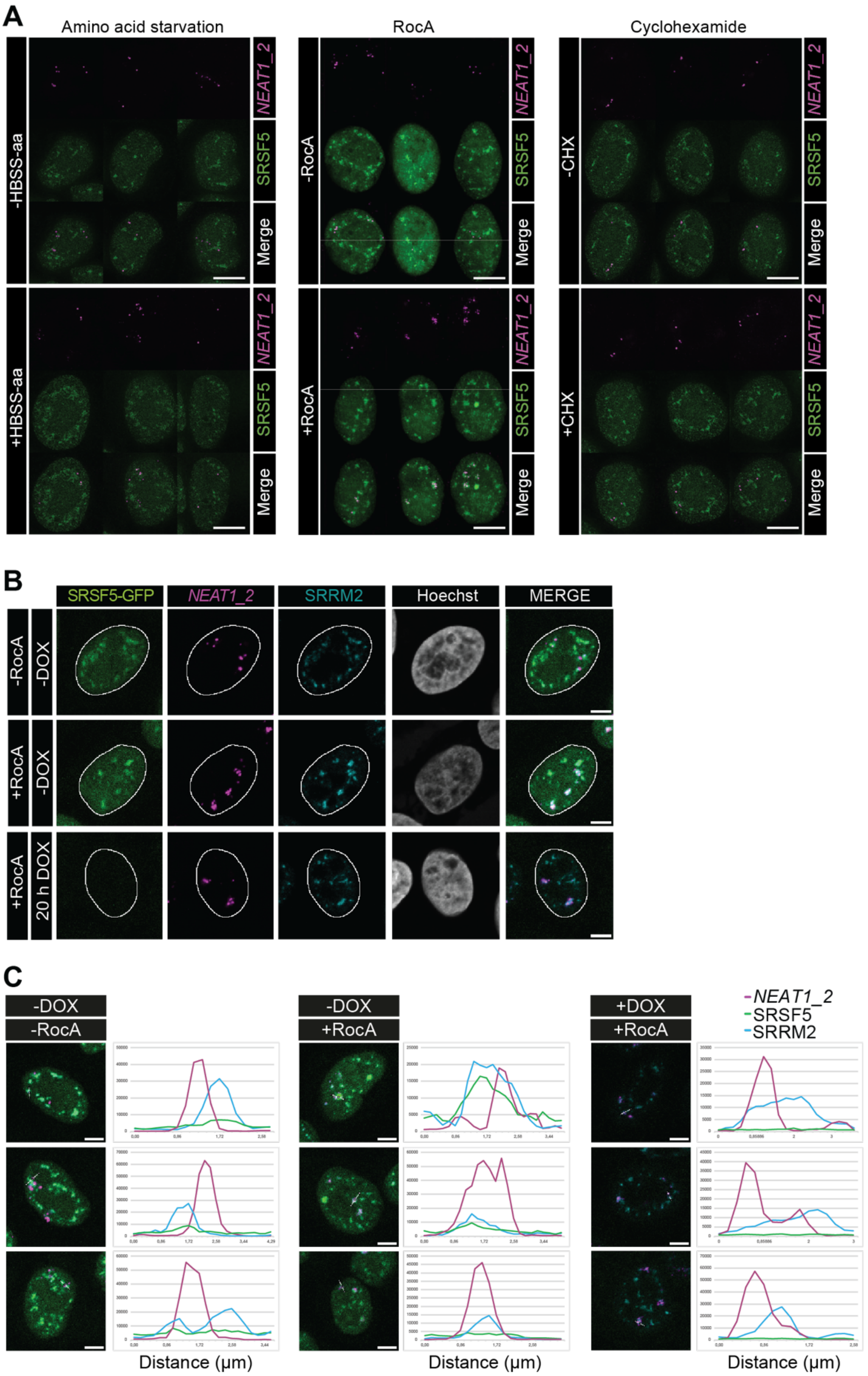

Figure S9: The merge between SRSF5 speckles and PS upon RocA treatment is not due to general translation inhibition. **A)** Example micrographs of SRSF5-GFP cells subjected to amino acid starvation (1 h), RocA treatment (4 h, 5 µM) and cyclohexamide (CHX) (1 h, 100 µg/mL) to inhibit translation. PS were labelled by RNA-FISH targeting the middle region of *NEAT1_2*. Scale bars - 8 µm. **B)** Example micrographs of SRSF5-GFP cells treated with RocA (4 h) and DOX (20 h, 1 µg/mL) to deplete SRSF5 simultaneously. **C)** Example line scans of SRSF5-GFP cells treated with RocA (4 h) and DOX (6 h) to monitor co-localization of SRSF5 signal, nuclear speckles and PS. PS were labelled by RNA-FISH targeting the middle region of *NEAT1_2*. NS are labelled by IF using an anti-SRRM2 antibody. Nuclei were stained with Hoechst. Scale bars - 5 µm.

Supplementary Tables

Table S1: List of proteins that bind to SRSF5 in close proximity on a short RNA fragment (partially RNase-resistant). Paraspeckle-relevant proteins are indicated based on (3).

Table S2: List of RNA FISH probes used in this study

Target	Fluorophore	Dilution	Supplier	Catalog-Nr.
NEAT1_2 middle	Quasar 670	125 nM in Hybridization buffer	BioCat	VSMF-2251-5-BS
NEAT1_2 5'end	Quasar 570	125 nM in Hybridization buffer	BioCat	VSMF-3034-5-BS
NEAT1_2 5'end	Quasar 670	125 nM in Hybridization buffer	BioCat	VSMF-2246-5-BS

Table S3: List of RNA FISH and DNA-PAINT strands for super resolution microscopy

Name	Figure	Sequences 5' to 3'	Supplier
**5'-dSTORM-Cy5**	**3ABC, S2E, 4CDE, 3G**	tgcggatattttccatgcag-Cy5<br>caagttgaagattagccctc-Cy5<br>agcccttggtctggaaaaaa-Cy5	Eurofins
**3'-dSTORM-Cy5**	**3F, S2D**	taaaggcatagccaggggac-Cy5<br>aaaagaaacacctgcggcgg-Cy5<br>aaaacctgagtgcggccatg-Cy5	Eurofins
**5'-DNA-PAINT-P1**	**3F**	tgcggatattttccatgcag-Docking P1<br>caagttgaagattagccctc-Docking P1<br>agcccttggtctggaaaaaa-Docking P1<br>aagttcagttccacaagacc-Docking P1<br>caggccgagcgaaaattaca-Docking P1<br>ctgtcaaacatgctaggtgc-Docking P1	Eurofins
**3'-inside-DNA-PAINT-P1**	**S2D**	aactgaacacgaggcacagt-Docking P1<br>ccaaggagcatgaagtcaga-Docking P1<br>cagcttcacatccacatgtc-Docking P1<br>taaaggcatagccaggggac-Docking P1<br>aaaagaaacacctgcggcgg-Docking P1<br>aaaacctgagtgcggccatg-Docking P1	Eurofins
Nanobody@**GFP**_LP1	**3G, S2E**	Docking LP1	NanoTag
5'-DNASTED-P1	**3E, S2C**	tgcggatattttccatgcag-Docking P1<br>caagttgaagattagccctc-Docking P1<br>agcccttggtctggaaaaaa-Docking P1<br>aagttcagttccacaagacc-Docking P1<br>caggccgagcgaaaattaca-Docking P1<br>ctgtcaaacatgctaggtgc-Docking P1<br>aagcgttggtcaatgttgtc-Docking P1<br>gtggagtgagctcacaagaa-Docking P1<br>cttaccagatgaccaggtaa-Docking P1<br>ttaccaacaataccgactcc-Docking P1<br>tgtaactttcagctggatgg-Docking P1<br>acaaaccacggtccatgaag-Docking P1<br>tcgccatgaggaacactata-Docking P1<br>atctgcaggcatcaattgag-Docking P1<br>agcaaggcctggaaacagaa-Docking P1<br>catctgctgtggacttttta-Docking P1	Eurofins

		ttattcaaagctgatggccc-Docking P1<br>ttcatgggctctggaacaag-Docking P1<br>gatgcagcatctgaaaacct-Docking P1<br>aaactagtatgaccggaggc-Docking P1	
3'-DNASTED-P5	**3E, S2C**	agactttttgccatatctga-Docking P5<br>cccaagaccttaatcattca-Docking P5<br>accttttctgtgcctctaa-Docking P5<br>catttcacattaagaaccca-Docking P5<br>agagacacactggagttgtg-Docking P5<br>tgtctgctgacattctacac-Docking P5<br>atgatccagcacatctagca-Docking P5<br>tcagaaatcacagagggact-Docking P5<br>tcaggcctaggaatagcttg-Docking P5<br>atttgctttacaagtcctgc-Docking P5<br>caaaacctgcctgatgatgc-Docking P5<br>tcacaagtacctgcacgata-Docking P5<br>agggacaggaaacagcagct-Docking P5<br>gtctagatatttcccatcat-Docking P5<br>cagtgcagagggttgggaac-Docking P5<br>cagctggtgttacaagaccc-Docking P5<br>agctcctaaaagaaggccag-Docking P5<br>taatagcccagtaaccctgg-Docking P5	Eurofins
Shell-DNASTED-P1/P5	**1B, 7ADEF, 8A**	tgcggatattttccatgcag-Docking P1/P5<br>caagttgaagattagccctc-Docking P1/P5<br>agcccttggtctggaaaaaa-Docking P1/P5<br>aagttcagttccacaagacc-Docking P1/P5<br>caggccgagcgaaaattaca-Docking P1/P5<br>ctgtcaaacatgctaggtgc-Docking P1/P5<br>aagcgttggtcaatgttgtc-Docking P1/P5<br>gtggagtgagctcacaagaa-Docking P1/P5<br>cttaccagatgaccaggtaa-Docking P1/P5<br>ttaccaacaataccgactcc-Docking P1/P5<br>tgtaactttcagctggatgg-Docking P1/P5<br>acaaaccacggtccatgaag-Docking P1/P5<br>tcgccatgaggaacactata-Docking P1/P5<br>atctgcaggcatcaattgag-Docking P1/P5<br>agcaaggcctggaaacagaa-Docking P1/P5<br>catctgctgtggacttttta-Docking P1/P5<br>ttattcaaagctgatggccc-Docking P1/P5<br>ttcatgggctctggaacaag-Docking P1/P5<br>gatgcagcatctgaaaacct-Docking P1/P5<br>aaactagtatgaccggaggc-Docking P1/P5<br>agactttttgccatatctga-Docking P1/P5<br>cccaagaccttaatcattca-Docking P1/P5<br>accttttctgtgcctctaa-Docking P1/P5<br>catttcacattaagaaccca-Docking P1/P5<br>agagacacactggagttgtg-Docking P1/P5<br>tgtctgctgacattctacac-Docking P1/P5<br>atgatccagcacatctagca-Docking P1/P5<br>tcagaaatcacagagggact-Docking P1/P5<br>tcaggcctaggaatagcttg-Docking P1/P5<br>atttgctttacaagtcctgc-Docking P1/P5	Eurofins

		caaaacctgcctgatgatgc-Docking P1/P5<br>tcacaagtacctgcacgata-Docking P1/P5<br>agggacaggaaacagcagct-Docking P1/P5<br>gtctagatatttcccatcat-Docking P1/P5<br>cagtgcagagggttgggaac-Docking P1/P5<br>cagctggtgttacaagaccc-Docking P1/P5<br>agctcctaaaagaaggccag-Docking P1/P5<br>taatagcccagtaaccctgg-Docking P1/P5	
Core-DNASTED-P5	**7AE, S6AB**	gttagtggctatgtagggaa-Docking P5<br>tttcacatggttagtggtca-Docking P5<br>gtgctttttgcaccaacaat-Docking P5<br>ctgatttttaaagtcctggt-Docking P5<br>ggagtacatacatgtttagc-Docking P5<br>gagaggagtgttcattgtaa-Docking P5<br>accaagacagtatttagtca-Docking P5<br>tattgactgacctggaacga-Docking P5<br>cctccatttaccagatttta-Docking P5<br>atattcttgccttacaggag-Docking P5<br>gtgtatttgagttccaacag-Docking P5<br>agattgagatttacccagtt-Docking P5<br>aaagagagccatgttgtgtc-Docking P5	Eurofins
Nanobody@GFP_P1	**1B, 7DEF, 8A, S1B, S9AB**	Docking P1	Massive Photonics
Docking LP1		Azide-ttatacatcta(left handed DNA)	biomers.net
Docking P1		ttatacatcta	biomers.net
**Docking P5**		tttcaatgtat	biomers.net
Imager LP1-AT655	**3G, S2E**	tagatgtat(left handed DNA)-ATTO 655	biomers.net
Imager P1-AT655	**3F, S2D**	tagatgtat -ATTO 655	Eurofins
**Imager P1-STAR635P**	**3E, S2C, 1B, 7ADEF, 8A, S1B, S9AB**	tagatgtat -Abberior STAR635P	biomers.net
**Imager P5-STAROrange**	**3E, S2C, 1B, 7ADEF, 8A, S6AB**	atacattga-Abberior STAROrange	biomers.net

Table S4: List of sgRNAs used in this study

Species	Name/Target	Protospacer sequence	PAM	Purpose	Supplier
**Human**	TARDBP_gRNA	gcuggggaauguagacagug	TCTG	NHEJ, GFP-tagging	Eurofins Genomics
**Human**	SRSF5_sg1 crRNA	tactagccggacatcatgag	TGG	NHEJ, SRSF5 KO	IDT
**Human**	SRSF5_sg2 crRNA	gatgctgtgtatgagcttga	TGG	NHEJ, SRSF5 KO	IDT
**-**	universal tracrRNA	-	-	-	IDT

Table S5: List of antibodies used in this study

Name	Species	Supplier	Catalog-Nr.
**α-GFP**	Goat	E. Geertsma, MPI-CBG	-
**α-INTS10**	Rabbit	Abcam	ab180934
**α-SRp40 (SRSF5)**	Rabbit	Merck Millipore	06-1365
**α-TDP-43**	Rabbit	Abcam	ab109535
**α-alpha-Tubulin**	Rabbit	Abcam	ab176560
**α-SRRM2**	Rabbit	ThermoFisher	PA5-59559
**α-G3BP1**	Mouse	Abcam	ab56574
**α-goat-HRP**	Donkey	Sigma-Aldrich	AB324P
**α-rabbit-HRP**	Donkey	Merck Millipore	AP182P
**α-rabbit-Alexa Fluor 680**	Donkey	ThermoFisher	A10043
**α-rabbit-Alexa Fluor Plus 405**	Donkey	ThermoFisher	A48258
**α-rabbit-Alexa Fluor Plus 555**	Goat	ThermoFisher	A32732
**α-rabbit-Alexa Fluor Plus 488**	Goat	ThermoFisher	A-11008
**α-mouse-Alexa Fluor Plus 594**	Donkey	ThermoFisher	A-21203
**α-rabbit-Alexa Fluor Plus 594**	Donkey	ThermoFisher	A-21207

Table S6: List of Cell lines used in this study

Name	Selection	Source	Species
**HeLa wild type**	-	ATCC	Human
**HeLa SRSF5 KO**	-	D. Stanek / This work	Human
**HeLa hGRAD NONO-GFP**	Puromycin/Geneticin	M. Müller-McNicoll (1)	Human
**HeLa hGRAD SRSF5-GFP**	Puromycin/Geneticin	M. Müller-McNicoll (1)	Human
**HeLa hGRAD SRRM2-GFP**	Puromycin/Geneticin	M. Müller-McNicoll (1)	Human
**HeLa hGRAD TDP43-GFP**	Hygromycin/Puromycin	S. Müller / This work	Human

Table S7: List of primers used in this study

Primer	Primer Sequence (5'-3')	Insert / Amplicon
qPCR_U6_F	gctcgcttcggcagc	*U6* snRNA qPCR Normalization
qPCR_U6_R	aaatatggaacgcttcacgaatt	*U6* snRNA qPCR Normalization
qPCR_hNEAT1_all_F	aggtctaggggggaccacagt	*NEAT1 all*
qPCR_hNEAT1_all_R	ctcagctatgcaagagcggc	*NEAT1 all*
qPCR_hNEAT1_2_F	tcttctaaagggccgccaca	*NEAT1_2*
qPCR_hNEAT1_2_R	gctgaggccagaggaactca	*NEAT1_2*
qPCR_TARDBP_E4/5_F	aggggtttggctttgttcgt	*TARDBP* exon4/5 junction
qPCR_TARDBP_E4/5_R	ggctcatcttggctttgctta	*TARDBP* exon4/5 junction
qPCR_TARDBP_E1/2_F	cggcctagcgggaaaagt	*TARDBP* exon1/2 junction
qPCR_TARDBP_E1/2_R	attcctgtagcgaagcccac	*TARDBP* exon1/2 junction
qPCR_INTS10_F	tgctgtacgacatgtttgtga	*INTS10*
qPCR_INTS10_R	atttcacactggacccgacc	*INTS10*
qPCR_INTS11_F	agtcacgcccttgggg	*INTS11*
qPCR_INTS11_R	ccaggtggaagtggctaatga	*INTS11*
Repair cassette M1_F	tggggatcagcatccaatgcaggg tcgggcagtggttttaatggaggc tttggctcaagcatggattctaag	*TARDBP* homology arms in exon 6, repair cassette containing eGFP and Puromycin resistance gene

	tcttctggctggggaatgtcaggt ggaggaggtagtg	
Repair cassette M2_R	tttagaaaaatttgaattcccacc attctataccaaccaaccacaacc ccactgtaaaaaacactgtctaca ttccccagcatctacacttagtag aaattagctagctgcatcggtacc	*TARDBP* homology arms in exon 6, repair cassette containing eGFP and Puromycin resistance gene

Table S8: List of plasmids used in this study

Name	Resistance	Source	Species
**pTRE-BI-hGRAD-mCherry**	Ampicillin Puromycin	M. Müller-McNicoll (1) Addgene #207837	Human
**P33_pEGFP-C1_hTDP43_WT**	Kanamycin	D. Dormann (4)	Human
**P38_pEGFP-C1_hTDP43_83_97AAA_Si65Res**	Kanamycin	D. Dormann (5)	Human
**pcDNA3.1-hAsCpf1(TYCV) (pY210)**	Ampicillin	F. Zhang (6), Addgene #89351	*Acidaminococcus* sp.
**pMaCTag-P05 repair cassette plasmid**	Ampicillin Puromycin	M. Knop (7), Addgene #120016	Human

Supplemental references

1. Arnold, B., Riegger, R.J., Okuda, E.K., Slišković, I., Keller, M., Bakisoglu, C., McNicoll, F., Zarnack, K. and Müller-McNicoll, M. (2024) hGRAD: A versatile "one-fits-all" system to acutely deplete RNA binding proteins from condensates. *J Cell Biol*, **223**.
2. Van Nostrand, E.L., Freese, P., Pratt, G.A., Wang, X., Wei, X., Xiao, R., Blue, S.M., Chen, J.Y., Cody, N.A.L., Dominguez, D. *et al.* (2020) A large-scale binding and functional map of human RNA-binding proteins. *Nature*, **583**, 711-719.
3. Yamazaki, T. and Hirose, T. (2015) The building process of the functional paraspeckle with long non-coding RNAs. *Front Biosci (Elite Ed)*, **7**, 1-41.
4. Bentmann, E., Neumann, M., Tahirovic, S., Rodde, R., Dormann, D. and Haass, C. (2012) Requirements for stress granule recruitment of fused in sarcoma (FUS) and TAR DNA-binding protein of 43 kDa (TDP-43). *J Biol Chem*, **287**, 23079-23094.
5. Gruijs da Silva, L.A., Simonetti, F., Hutten, S., Riemenschneider, H., Sternburg, E.L., Pietrek, L.M., Gebel, J., Dötsch, V., Edbauer, D., Hummer, G. *et al.* (2022) Disease-linked TDP-43 hyperphosphorylation suppresses TDP-43 condensation and aggregation. *EMBO J*, **41**, e108443.
6. Gao, L., Cox, D.B.T., Yan, W.X., Manteiga, J.C., Schneider, M.W., Yamano, T., Nishimasu, H., Nureki, O., Crosetto, N. and Zhang, F. (2017) Engineered Cpf1 variants with altered PAM specificities. *Nat Biotechnol*, **35**, 789-792.
7. Fueller, J., Herbst, K., Meurer, M., Gubicza, K., Kurtulmus, B., Knopf, J.D., Kirrmaier, D., Buchmuller, B.C., Pereira, G., Lemberg, M.K. *et al.* (2020) CRISPR-Cas12a-assisted PCR tagging of mammalian genes. *J Cell Biol*, **219**.
